# *Campylobacter* Abundance in Breastfed Infants and Identification of a New Species in the Global Enterics Multicenter Study

**DOI:** 10.1128/mSphere.00735-19

**Published:** 2020-01-15

**Authors:** Xiaoming Bian, Jolene M. Garber, Kerry K. Cooper, Steven Huynh, Jennifer Jones, Michael K. Mills, Daniel Rafala, Dilruba Nasrin, Karen L. Kotloff, Craig T. Parker, Sharon M. Tennant, William G. Miller, Christine M. Szymanski

**Affiliations:** a Complex Carbohydrate Research Center, University of Georgia, Athens, Georgia, USA; b Department of Microbiology, University of Georgia, Athens, Georgia, USA; c School of Animal and Comparative Biomedical Sciences, University of Arizona, Tucson, Arizona, USA; d Produce Safety and Microbiology Research Unit, Agricultural Research Service, U.S. Department of Agriculture, Albany, California, USA; e Center for Vaccine Development and Global Health, University of Maryland School of Medicine, Baltimore, Maryland, USA; University of Michigan—Ann Arbor

**Keywords:** *Campylobacter*, breastfeeding, GEMS, l-fucose metabolism, “*Candidatus* Campylobacter infans,” gut microbiome

## Abstract

Campylobacter jejuni is a leading cause of bacterial diarrhea worldwide and is associated with high rates of mortality and growth stunting in children inhabiting low- to middle-resource countries. To better understand the impact of breastfeeding on *Campylobacter* infection in infants in sub-Saharan Africa and South Asia, we examined fecal microbial compositions, bacterial isolates, and their carbohydrate metabolic pathways in *Campylobacter*-positive infants <1 year of age from the Global Enterics Multicenter Study. Exclusively breastfed infants with diarrhea exhibited high *Campylobacter* abundances, and this negatively correlated with bacterial carbohydrate metabolism. Although C. jejuni and Campylobacter coli are prevalent among these infants, the second most abundant *Campylobacter* species was a new species, which we named “*Candidatus* Campylobacter infans.” Asymptomatic *Campylobacter* carriers also possess significantly different proportions of specific gut microbes compared to diarrheal cases. These findings provide insight into *Campylobacter* infections in infants in sub-Saharan Africa and South Asia and help inform strategies aimed at eliminating campylobacteriosis in these areas.

**IMPORTANCE** *Campylobacter* is the primary cause of bacterial diarrhea in the United States and can lead to the development of the postinfectious autoimmune neuropathy known as Guillain-Barré syndrome. Also, drug-resistant campylobacters are becoming a serious concern both locally and abroad. In low- and middle-income countries (LMICs), infection with *Campylobacter* is linked to high rates of morbidity, growth stunting, and mortality in children, and breastfeeding is important for infant nutrition, development, and protection against infectious diseases. In this study, we examined the relationship between breastfeeding and *Campylobacter* infection and demonstrate the increased selection for C. jejuni and C. coli strains unable to metabolize fucose. We also identify a new *Campylobacter* species coinfecting these infants with a high prevalence in five of the seven countries in sub-Saharan Africa and South Asia examined. These findings indicate that more detailed studies are needed in LMICs to understand the *Campylobacter* infection process in order to devise a strategy for eliminating this pathogenic microbe.

## Abstract (precis)

*Campylobacter* is the primary cause of bacterial diarrhea in the United States and can lead to the development of the postinfectious autoimmune neuropathy known as Guillain-Barré syndrome. Also, drug-resistant campylobacters are becoming a serious concern both locally and abroad. In low- and middle-income countries (LMICs), infection with *Campylobacter* is linked to high rates of morbidity, growth stunting, and mortality in children, and breastfeeding is important for infant nutrition, development, and protection against infectious diseases. In this study, we examined the relationship between breastfeeding and *Campylobacter* infection and demonstrate the increased selection for C. jejuni and C. coli strains unable to metabolize fucose. We also identify a new *Campylobacter* species coinfecting these infants with a high prevalence in five of the seven countries in sub-Saharan Africa and South Asia examined. These findings indicate that more detailed studies are needed in LMICs to understand the *Campylobacter* infection process in order to devise a strategy for eliminating this pathogenic microbe.

## INTRODUCTION

Campylobacter jejuni is one of the major causes of bacterial diarrhea worldwide, and infection leads to a high rate of childhood mortality in low- to middle-income countries (LMICs) (1, 2). Campylobacteriosis is characterized by watery or bloody diarrhea, fever, and abdominal pain and can persist from a few days to several weeks. Occasionally, serious postinfectious sequelae, such as Guillain-Barré syndrome, irritable bowel syndrome, and reactive arthritis, can follow diarrheal disease (3). In LMICs, children under 2 years of age are particularly susceptible to *Campylobacter*-induced diarrhea, and asymptomatic carriage of *Campylobacter* species in healthy children is also common (3). C. jejuni can disrupt intestinal tight junctions and induces gut leakage and systemic inflammation (4, 5). Both symptomatic and asymptomatic *Campylobacter* infections are reported to be associated with growth stunting in children (6, 7).

The *Campylobacter* genus contains 31 species (http://www.bacterio.net/campylobacter.html), many of which are known pathogens in humans (8). C. jejuni and Campylobacter coli are among the most commonly detected species associated with gastroenteritis, but emerging *Campylobacter* species, such as Campylobacter upsaliensis, Campylobacter lari, and Campylobacter hyointestinalis, are receiving more attention due to their increasing association with human diseases, likely resulting from improved isolation and detection techniques (8). For example, some *Campylobacter* spp., including C. hyointestinalis and Campylobacter fetus, are sensitive to antimicrobial agents used in traditional *Campylobacter*-selective agar and are isolated more frequently using the Cape Town protocol with antibiotic-free blood agar and H_2_ enrichment (9).

Contaminated foods and water are considered the main sources of *Campylobacter* infections in LMICs, and the risk of *Campylobacter*-associated diarrhea in children has been suggested to be reduced by several factors, including breastfeeding (7). In infants <1 year of age, the mother’s milk not only provides nutrition but also supports the establishment of a healthy gut microbiota and the development of a robust immune system (10). Human milk contains antibodies against various pathogens, including *Campylobacter*, and human milk oligosaccharides (HMOs), particularly fucosyloligosaccharides such as 2′-fucosyllactose, have been reported to inhibit some C. jejuni strains from binding to intestinal epithelial cells and colonizing mouse models (11, 12). Also, HMOs stimulate the growth of beneficial bacteria, such as *Bifidobacterium*, which provide a variety of health benefits, including competition with enteric pathogens such as *Campylobacter* (13).

Children who are not breastfed do not receive protective antibodies or HMOs and are thus at a higher risk for infection, particularly diarrheal disease (3, 14). Yet 85% of children had *Campylobacter*-positive stools by 1 year of age in LMICs where breastfeeding is common (7, 15). Our previous studies have shown that >50% of sequenced C. jejuni and C. coli isolates possess a *fuc* locus (*cj0480c* to *cj0489*) (16) for l-fucose metabolism, providing strains such as C. jejuni NCTC 11168 with a competitive advantage in the colonization of a piglet model for diarrheal disease (16, 17). l-Fucose can be released from fucosylated HMOs by several gut microbes, including *Bacteroides* and *Bifidobacterium* species (18–20); thus, it is possible that human milk may actually provide a rich carbon source to promote the growth of *Campylobacter* strains capable of metabolizing l-fucose. This hypothesis had not been previously explored since C. jejuni and C. coli were considered to be asaccharolytic, relying solely on amino acids and tricarboxylic acid (TCA) cycle intermediates for carbon sources (21). Now, with the discovery that many *Campylobacter* isolates metabolize l-fucose, together with the observation that free l-fucose levels can reach 4 to 5 mg/g feces in breastfed children (22) and reports that d-glucose is also abundant in feces of breastfed children (22) and that ∼1.7% of sequenced C. jejuni and C. coli strains possess the Entner-Doudoroff (ED) pathway for glucose utilization (23), we sought to examine the relationship between breastfeeding and *Campylobacter* infections to better understand the process of *Campylobacter*-induced diarrhea in infants.

To measure the burden and bacterial etiology of diarrheal disease in children under 5 years of age in LMICs, the Global Enterics Multicenter Study (GEMS) was conducted in seven countries in sub-Saharan Africa (The Gambia, Kenya, Mali, and Mozambique) and South Asia (Bangladesh, India, and Pakistan) (24). Liu et al. found that together with other common pathogens, C. jejuni and C. coli were major causes of diarrhea in this age group (2). To examine the relationship between *Campylobacter* infection and breastfeeding in infants <1 year of age in the GEMS data set at a molecular level, we applied molecular techniques to examine the relative abundances of campylobacters and other resident gut microbes in asymptomatic and symptomatic *Campylobacter* infections. We also screened the C. jejuni and C. coli isolates for their ability to utilize l-fucose and d-glucose and assessed whether this correlated with diarrheal disease. Through these studies, we identified a new *Campylobacter* species that is present in stool samples of infants from multiple countries and is as prevalent as C. coli.

## RESULTS

### Data description.

Our study included a total of 233 fecal DNA samples from symptomatic and asymptomatic *Campylobacter*-infected infants <1 year of age that were collected at seven sites (The Gambia, Kenya, Mali, Mozambique, Bangladesh, India, and Pakistan), with infants either exclusively or not breastfed (see Fig. S1 and Table S1 in the supplemental material). Of these 233 samples, 128 were from symptomatic infections (cases) and 105 were from asymptomatic infections (controls); 142 infants were exclusively breastfed, while 91 were not breastfed at the time of stool collection; 110 were 0 to 5 months old, while 123 were 6 to 11 months old; and 111 had moderate-to-severe diarrhea (MSD), while 17 had less-severe diarrhea (LSD).

10.1128/mSphere.00735-19.1 FIG S1 Flow diagram illustrating study activities. Download FIG S1, TIF file, 1.3 MB. Copyright © 2020 Bian et al. 2020 Bian et al. This content is distributed under the terms of the Creative Commons Attribution 4.0 International license.

10.1128/mSphere.00735-19.6 TABLE S1 Characteristics of study subjects with symptomatic or asymptomatic *Campylobacter* infections. Download Table S1, DOCX file, 0.01 MB. Copyright © 2020 Bian et al. 2020 Bian et al. This content is distributed under the terms of the Creative Commons Attribution 4.0 International license.

In 84.12% of the infants, comorbidities with other diarrheal pathogens were identified (Fig. S2), including viruses (astrovirus, norovirus, adenovirus, rotavirus, and sapovirus), other bacteria (*Aeromonas*, Bacteroides fragilis, Clostridium difficile, Escherichia coli, Helicobacter pylori, *Shigella*, *Salmonella* [nontyphoidal], and *Vibrio*), and parasites (*Giardia*, *Entamoeba*, and *Cryptosporidium*). Comorbidities with viruses were found in 33.48% of the infants, comorbidities with other bacteria were found in 66.95% of the infants, and comorbidities with parasites were found in 22.75% of the infants.

10.1128/mSphere.00735-19.2 FIG S2 Coinfection with other diarrheal pathogens, including viruses (astrovirus, norovirus, adenovirus, rotavirus, and sapovirus), other bacteria (*Aeromonas*, Bacteroides fragilis, Clostridium difficile, Escherichia coli, Helicobacter pylori, *Shigella*, *Salmonella* [nontyphoidal], and *Vibrio*), and parasites (*Giardia*, *Entamoeba*, and *Cryptosporidium*), in addition to *Campylobacter*. Percent values are the proportions in *Campylobacter*-infected infants. Download FIG S2, TIF file, 0.8 MB. Copyright © 2020 Bian et al. 2020 Bian et al. This content is distributed under the terms of the Creative Commons Attribution 4.0 International license.

The V6-V8 hypervariable region of the 16S rRNA gene was amplified from 229 samples and sequenced using the Illumina MiSeq platform at the University of Georgia Genomics and Bioinformatics Core; 4 of the 233 samples did not have amplification products and were not sequenced, and all 4 had MSD, with exclusive breastfeeding (2 samples) or no breastfeeding (2 samples). Of the 229 sequenced samples, 140 were from infants who were exclusively breastfed (diarrhea, *n* = 75; no diarrhea, *n* = 65), and 89 were from infants who were not breastfed (diarrhea, *n* = 49; no diarrhea, *n* = 40) at the time of stool collection. A total of 2,899 different amplicon sequence variants (ASVs) were obtained, with a median of 22,244 sequences per sample. Of the 229 samples, a random subset of 67 was amplified using the V4 hypervariable region of the 16S rRNA gene for additional confirmation.

A total of 142 C. jejuni and C. coli strains isolated from the fecal samples (1 isolate per sample) were tested for l-fucose utilization (24, 25). Of these 142 strains, 80 were from symptomatic infants (exclusively breastfed, *n* = 51; nonbreastfed, *n* = 29), and 62 were from asymptomatic infants (exclusively breastfed, *n* = 32; nonbreastfed, *n* = 30).

### Compositional variations of fecal microbiota in cases and controls.

**FIG 1:**
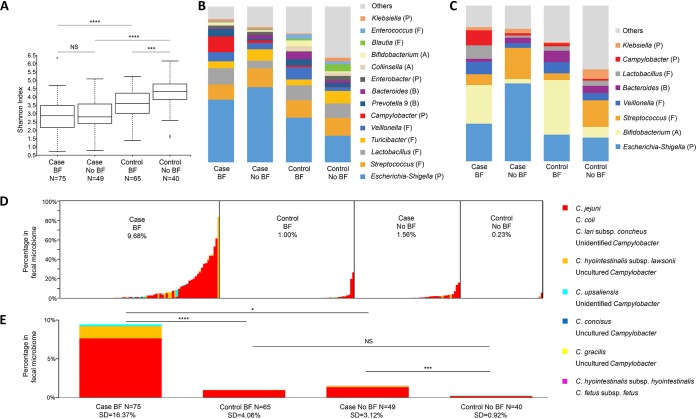
Fecal microbiome in symptomatic and asymptomatic *Campylobacter* infections in infants <1 year of age. (A) Microbial diversities (Shannon index) in different groups. Center line, median; box limits, upper and lower quartiles; whiskers, 1.5× interquartile range; points, outliers. (B) Microbial compositions of fecal samples from cases and controls with breastfeeding (BF) or no breastfeeding (No BF) (V6-V8 of 16S rRNA). P, F, A, and B refer to phyla (P, *Proteobacteria*; F, *Firmicutes*; A, *Actinobacteria*; B, *Bacteroidetes*). (C) Microbial composition of fecal samples from cases and controls with breastfeeding or no breastfeeding (V4 of 16S rRNA). (D) Proportions of *Campylobacter* species at the individual-child level. Each of the bars represents one individual subject, and different *Campylobacter* species are shown in different colors. (E) Average *Campylobacter* abundance in each group. NS, not statistically significant; *, *P* < 0.05; ***, *P* < 0.001; ****, *P* < 0.0001.

The Shannon index was calculated to measure the fecal microbial diversity (Fig. 1A) and showed that infants with diarrhea, with or without breastfeeding, had significantly lower fecal microbial diversity (*P < *0.01) than asymptomatic infants. Nonbreastfed asymptomatic infants demonstrated the highest fecal diversity (*P < *0.001), with fecal diversity within the exclusively breastfed asymptomatic infants being intermediate between the two groups (*P < *0.01).

The proportional abundance of taxa comprising each fecal microbiome was determined by sequencing the 16S rRNA V6-V8 hypervariable regions from each sample (Fig. 1B). This analysis showed that *Proteobacteria* were the most abundant in infants with diarrhea, while the *Firmicutes* were the most abundant in the controls (Fig. S3A and B). *Escherichia* and *Shigella* were the most abundant in all groups regardless of diarrheal status and breastfeeding; however, in infants who were not breastfed, the abundance of *Escherichia-Shigella* in cases (48.1%) was significantly higher than in controls (17.0%), while in exclusively breastfed infants, the difference was not statistically significant (40.1% in cases and 28.6% in controls) (Table S2). Genera with significant differences between cases and controls are shown in Table S2.

10.1128/mSphere.00735-19.3 FIG S3 Fecal microbiome in symptomatic and asymptomatic *Campylobacter* infections of infants <1 year of age. (A) Microbial composition of fecal samples in cases and controls with or without breastfeeding at the phylum level (V6-V8 of 16S rRNA). (B) Microbial composition of fecal samples in cases and controls with or without breastfeeding at the phylum level (V4 of 16S rRNA). (C to J) Proportion of *Campylobacter* in the fecal microbiome at the individual-child level and average in each country as determined by 16S rRNA sequencing (V6-V8 regions). Each of the bars represents one individual, and different *Campylobacter* species are shown in the colors indicated. (C to F) Individual levels per country. (G to J) Averages per country. BD, Bangladesh; GM, The Gambia; KE, Kenya; MZ, Mozambique. (K and L) Proportion of *Campylobacter* in the fecal microbiome at the individual level in different age groups (0 to 5 months [K] and 6 to 11 months [L]). (I) Proportions of *Campylobacter* in fecal microbiomes between dysenteric and nondysenteric diarrheal cases. Download FIG S3, TIF file, 1.4 MB. Copyright © 2020 Bian et al. 2020 Bian et al. This content is distributed under the terms of the Creative Commons Attribution 4.0 International license.

10.1128/mSphere.00735-19.7 TABLE S2 Significantly different genera between cases and controls. Download Table S2, DOCX file, 0.01 MB. Copyright © 2020 Bian et al. 2020 Bian et al. This content is distributed under the terms of the Creative Commons Attribution 4.0 International license.

The V4 hypervariable region of the 16S rRNA gene amplifies *Bifidobacterium* effectively (26, 27), and a higher proportion of *Bifidobacterium* in exclusively breastfed than in nonbreastfed infants was observed following sequencing of the V4 hypervariable region (Fig. 1C). The *Bifidobacterium* genus is the most abundant in exclusively breastfed infants, but a higher abundance of *Escherichia-Shigella* was still found in cases (50.01%) than in controls (15.22%) with no breastfeeding (not statistically significant due to the smaller sample size of the V4 data set).

The relative abundance of *Campylobacter* was negatively correlated with several gut microbes, including *Dorea*, *Blautia*, *Klebsiella*, *Erysipelatoclostridium*, *Enterobacter*, *Faecalibacterium*, *Enterococcus*, *Collinsella*, the Ruminococcus gnavus group, *Lactococcus*, and *Anaerostipes*, and these gut microbes were positively correlated with each other (Table S3).

10.1128/mSphere.00735-19.8 TABLE S3 Correlations between *Campylobacter* and gut microbes. Download Table S3, DOCX file, 0.01 MB. Copyright © 2020 Bian et al. 2020 Bian et al. This content is distributed under the terms of the Creative Commons Attribution 4.0 International license.

### Relative abundance of *Campylobacter* in symptomatic and asymptomatic infants.

Although *Campylobacter* strains were isolated from all the fecal samples included in our study, 16S rRNA sequencing (V6-V8 regions) of the fecal DNA detected a fraction of them (cases with exclusive breastfeeding, 61/75; controls with exclusive breastfeeding, 31/65; cases with no breastfeeding, 34/49; controls with no breastfeeding, 10/40). As shown in Fig. 1D and E, the 16S rRNA of *Campylobacter* had >0.99 identity value to C. jejuni, C. coli, C. lari, C. hyointestinalis, C. upsaliensis, and other campylobacters. A greater abundance of *Campylobacter* species was found in cases with breastfeeding (∼10%) than in other groups (Fig. 1B). Consistent with results obtained from sequencing the V6-V8 rRNA regions, the results with the V4 regions (Fig. 1C) showed that *Campylobacter* species are also more abundant in cases with breastfeeding (∼10%) than in all other groups (not statistically significant because of the smaller V4 sample size).

The relative abundance of *Campylobacter* in the fecal microbiome was highly variable in each group (Fig. 1D). In cases with breastfeeding, the highest abundance of *Campylobacter* was in one child with 83% of the fecal microbiome comprised of one strain with 0.99 identity value to C. hyointestinalis subsp. *lawsonii*/uncultured *Campylobacter* by 16S rRNA sequencing. Our results clearly indicate that exclusively breastfed infants with diarrheal symptoms had statistically the highest abundance of campylobacters compared to other groups, including nonbreastfed cases (Fig. 1E). Among nonbreastfed infants, cases had higher levels of *Campylobacter* than controls (Fig. 1E). When comparing controls with and those without breastfeeding, exclusively breastfed infants had higher *Campylobacter* levels than nonbreastfed infants, but the results were not statistically significant (Fig. 1E). Similar trends were found when the data were examined for different sites and ages (Fig. S3C to H).

### Fucose metabolism in *Campylobacter* strains isolated from GEMS.

**FIG 2:**
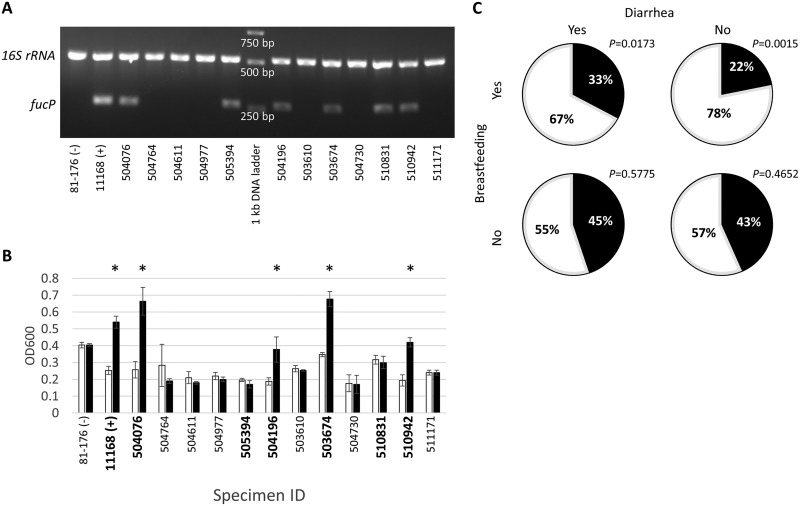
Investigation of the relationship between fucose metabolism and *Campylobacter* infection in GEMS. (A) A PCR screen for the fucose permease gene *fucP* was carried out for all samples, and a representative gel is shown. (B) Verification of the *fucP* colony PCR results to compare the abilities to metabolize fucose and show growth enhancement. Values shown are means (*n* = 3), and error bars represent 1 standard deviation (*fucP*^+^ strains are highlighted in boldface type). White, control; black, l-fucose addition. (C) Pie charts comparing the correlations between fucose metabolism and the isolated *Campylobacter* strains. Black, l-fucose-utilizing strains; white, non-l-fucose-utilizing strains. *P* values were determined by chi-squared testing compared to 50%.

Despite previous research suggesting that breastfeeding protects against C. jejuni-induced diarrhea (11, 28), reports from studies such as GEMS and MAL-ED indicated that a large proportion of breastfed children with diarrhea were *Campylobacter* positive, and our results unexpectedly showed even higher proportions of this genus in breastfed infants than in nonbreastfed infants. C. jejuni and C. coli were isolated in the original GEMS using selective plates for these two species (25). To assess whether there were any correlations between C. jejuni and C. coli abundances and carbohydrate metabolism, PCR screening for the fucose permease gene *fucP* was done. We used nested colony PCR for the *fucP* gene (with the 16S rRNA gene as a control) to screen for strains with the operon for fucose metabolism (Fig. 2A). The subset of isolates that were PCR positive was subsequently tested in growth assays in limited growth medium supplemented with l-fucose to confirm that they possessed a functional pathway (Fig. 2B). The results indicate that in nonbreastfed infants, approximately 50% of the strains were fucose utilizing versus nonutilizing (*P = *0.5775 and *P = *0.4652 for cases and controls, respectively), but in breastfed infants, significantly fewer fucose-utilizing strains (33% and 22% in cases and controls, respectively; *P < *0.05) were found (Fig. 2C). Previous studies suggested that there is a negative correlation between the possession of the fucose metabolic locus and the γ-glutamyl transpeptidase (GGT) gene (*ggt*), which provides the strain the ability to utilize glutamine and glutathione (29). Since glutamine is one of the most abundant free amino acids in breast milk (30), we screened for the *ggt* gene in all strains and found that only 15 of 137 (11%) screened strains possessed the *ggt* gene, 12 of 79 (15%) in strains lacking *fucP* and 3 of 58 (5%) *fucP^+^* strains. For the utilization of d-glucose, 25% of strains were randomly selected and tested in growth assays using limited growth medium supplemented with d-glucose, and none of the tested strains showed enhanced growth in glucose.

### A putative new *Campylobacter* species, “*Candidatus* Campylobacter infans.”

**FIG 3:**
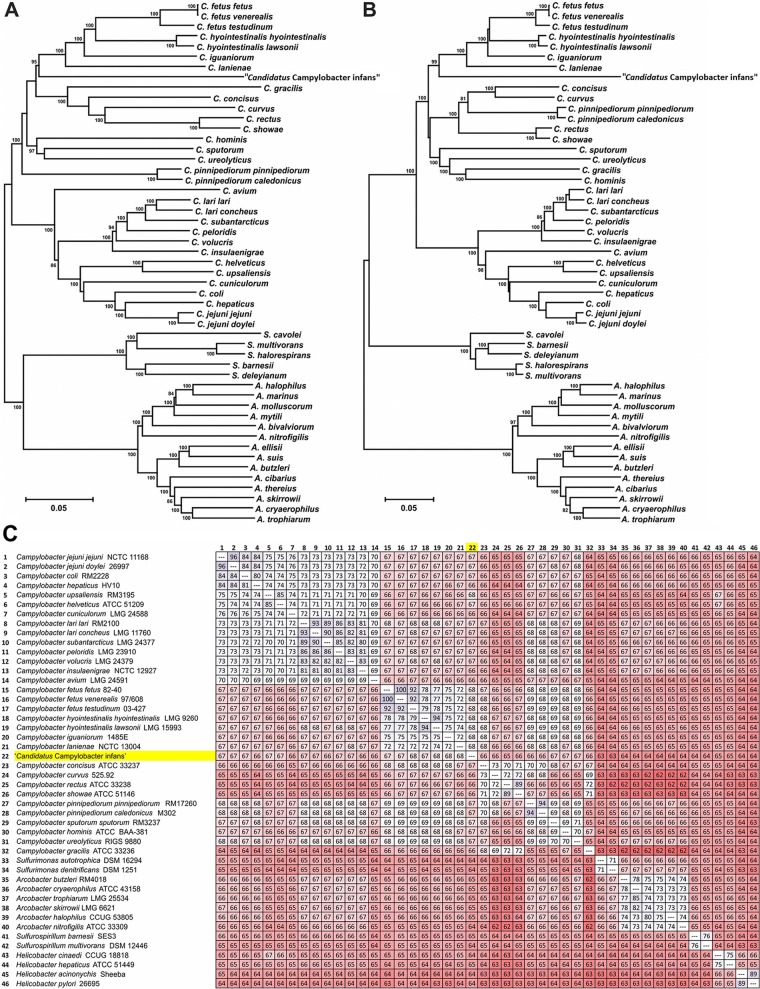
Identification of a new *Campylobacter* species. (A and B) Sequences of 20 core genes from various *Campylobacteraceae* or their cognate proteins were concatenated and aligned using CLUSTALX. Included in the alignment were the same 20 concatenated genes or proteins extracted from the metagenomic sequences obtained from a fecal DNA sample containing 83% *Campylobacter* sequences (“*Candidatus* Campylobacter infans”). The dendrograms were constructed using the neighbor-joining algorithm and the Kimura two-parameter (gene set) (A) or Poisson (protein set) (B) distance estimation method. Bootstrap values of >75%, generated from 500 replicates, are shown at the nodes. (C) Average nucleotide identity (ANI) of “*Candidatus* Campylobacter infans” among known related bacteria, including *Campylobacter*, *Sulfurimonas*, *Arcobacter*, *Sulfurospirillum*, and *Helicobacter* species.

Multiplex PCR of the lipid A biosynthesis gene *lpxA* was used to confirm the presence of C. jejuni, C. coli, and C. upsaliensis identified by 16S rRNA sequencing (31). However, no molecular identification method was available for C. hyointestinalis, and only one C. hyointestinalis subsp. *lawsonii* genome was present in GenBank (accession number CP015576), so we sequenced nine additional C. hyointestinalis subsp. *lawsonii* strains (32), and together with the genome of C. hyointestinalis subsp. *hyointestinalis* (GenBank accession number NZ_CP015575.1), we designed a primer specific for the C. hyointestinalis *lpxA* gene of both subspecies to be used for multiplex PCR. The revised *lpxA* multiplex PCR was performed on the known C. hyointestinalis strains (Fig. S4A) and applied to the GEMS fecal DNA samples that were putatively designated to belong to this species by 16S rRNA sequencing (Fig. S4B). Only samples G9, G21, and G22 have the expected 285-bp product out of the predicted 26 GEMS fecal samples and were confirmed to contain C. hyointestinalis, suggesting that the other campylobacters identified as C. hyointestinalis subsp. *lawsonii*/uncultured *Campylobacter* by 16S rRNA sequencing may actually be a new species. One of the 26 infants (G1) (Fig. S4C) had prolonged diarrhea for 9 days, and 83.6% of the fecal microbiome was comprised of this species, with no other *Campylobacter* species being detected by 16S rRNA analysis, although one C. jejuni strain was isolated from the fecal sample. Metagenomic sequencing of this fecal DNA sample was performed, and assembly of the Illumina reads yielded 6,058 total contigs and 75 contigs identified as *Campylobacter*, of which 56 were larger than 5,000 bp. Overall, the metagenomic analysis of the fecal sample found the dominant clusters of orthologous groups (COGs) to be associated with functions in metabolism, information storage and processing, and cellular processes and signaling, and in particular, 10.3% of sequence reads were associated with protein metabolism (Fig. S5A and B). While 16S rRNA sequencing found a higher percentage of the fecal microbiome composition to be *Campylobacter* (83.6%), metagenomic analysis confirmed at the order level (74.3%) and genus level (66.6%) that it was the dominant member of the microbial community (Fig. S5C to E). Furthermore, 33.8% of all the sequence data that were assembled into contigs particularly >10 kb were identified as *Campylobacter* (Fig. S5F), with an average of 76× coverage for these contigs, which further demonstrated that it was the major genus present in the fecal microbiome and provided sufficient coverage for genome assembly. BLASTP analysis of the 1,476 putative coding sequences identified in the 75 *Campylobacter* contigs indicated that the *Campylobacter* species present in this fecal sample was not one of the current validly described *Campylobacter* taxa; however, 72.8% (1,074/1,476) of the matches showed strong similarity to proteins from members of the C. fetus group (i.e., C. fetus, C. hyointestinalis, C. lanienae, and C. iguaniorum). Data from core gene/protein phylogenetic analyses (Fig. 3A and B) and average nucleotide identities (ANI) (Fig. 3C) are consistent with these results, suggesting that the *Campylobacter* strain in this fecal sample (referred to here as “*Candidatus* Campylobacter infans”) represents a novel *Campylobacter* species (Fig. 3) that is related to, but distinct from, C. fetus, C. hyointestinalis, *C. lanienae*, and *C. iguaniorum*.

10.1128/mSphere.00735-19.4 FIG S4 (A) Multiplex PCR to differentiate *Campylobacter* species using the *lpxA* gene. Lanes: 1, mixture of C. coli, C. jejuni, C. hyointestinalis (both subspecies), C. lari subsp. *lari*, and C. upsaliensis, with C. fetus as a control; 2, C. coli (391 bp); 3, C. jejuni (331 bp); 4, C. hyointestinalis subsp*. hyointestinalis* (285 bp); 5, C. hyointestinalis subsp. *lawsonii* (285 bp); 6, C. lari (233 bp); 7, C. upsaliensis (206 bp); 8, C. fetus (no product); 9 DNA ladder; 10 to 16, C. hyointestinalis subsp. *lawsonii* strains (285 bp). (B) PCR of the C. hyointestinalis *lpxA* gene from 26 GEMS fecal DNA samples possibly containing C. hyointestinalis. (C) *lpxA* gene PCR designed against the new “*Candidatus* Campylobacter infans” fecal metagenome strain with the 26 GEMS fecal DNA samples tested in panel B. Chl, C. hyointestinalis subsp. *lawsonii*; Chh, C. hyointestinalis subsp. *hyointestinalis*; G1, “*Candidatus* Campylobacter infans”; G2, 202699; G3, 203886; G4, 700541; G5, 302411; G6, 302176; G7, 700488; G8, 702610; G9, 702562; G10, 702298; G11, 700921; G12, 503818; G13, 700209; G14, 310426; G15, 703664; G16, 103067; G17, 110498; G18, 702556; G19, 704137; G20, 700270; G21, 700207; G22, 700173; G23, 100795; G24, 704231; G25, 704029; G26, 710603; NTC, no-template control. The first number of the 6-digit code indicates sample site (1, The Gambia; 2, Mali; 3, Mozambique; 5, India; 7, Pakistan). Download FIG S4, TIF file, 2.0 MB. Copyright © 2020 Bian et al. 2020 Bian et al. This content is distributed under the terms of the Creative Commons Attribution 4.0 International license.

10.1128/mSphere.00735-19.5 FIG S5 (A) Functional categories for clusters of orthologous groups (COGs) in metagenomic sequencing. Percentages are based on the number of reads with predicted function. (B) Functional categories for subsystems in metagenomics. Percentages are based on the number of reads with predicted subsystem function. (C) Taxonomic abundance at the genus level. Abundances are based on the number of reads for each genus. (D) Taxonomic distribution in metagenomic samples at the order level. Percentages are based on the number of reads for each order. (E) Taxonomic distribution in metagenomic samples at the genus level. Percentages are based on the number of reads for each genus. (F) Taxonomic distribution of total metagenomic sequencing. Percentages are based on the amount of sequences of the assembled contigs. Download FIG S5, TIF file, 1.8 MB. Copyright © 2020 Bian et al. 2020 Bian et al. This content is distributed under the terms of the Creative Commons Attribution 4.0 International license.

**FIG 4:**
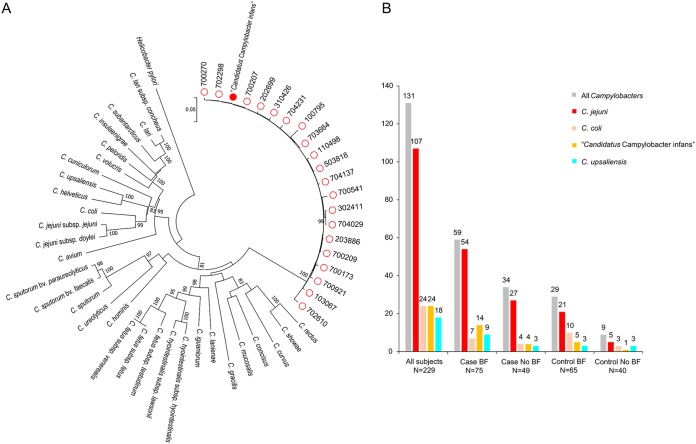
Detection of “*Candidatus* Campylobacter infans” and prevalences of different *Campylobacter* species. (A) Phylogenetic analysis of “*Candidatus* Campylobacter infans” in GEMS samples using the full *atpA* gene. Red circles, *atpA* from GEMS fecal DNA (the first number of the 6-digit code indicates sample site [1, The Gambia; 2, Mali; 3, Mozambique; 5, India; 7, Pakistan]); red solid circle, “*Candidatus* Campylobacter infans” *atpA.* The dendrogram was constructed using the neighbor-joining algorithm and the Tamura three-parameter method. Bootstrap values of >75%, generated from 1,000 replicates, are shown at the nodes. (B) Prevalences of different *Campylobacter* species in the study subjects, detected by 16S rRNA sequencing and confirmed by *lpxA* multiplex PCR and *atpA* sequencing (*atpA* sequencing was done only for “*Candidatus* Campylobacter infans”).

Using the metagenomic sequencing data, we designed *lpxA* primers for “*Candidatus* Campylobacter infans” for comparison with the *lpxA* primers used for the multiplex PCR that we performed previously and tested all 26 fecal samples that had 16S rRNA predictions closest to C. hyointestinalis subsp. *lawsonii*/uncultured *Campylobacter*. The PCR results indicated that 18 of the 26 samples had the expected PCR product, except samples G8, G9, G19, G20, G24, G25, and G26 (Fig. S4C). For further confirmation, specific primers for the “*Candidatus* Campylobacter infans” full-length *atpA* gene were designed and used to amplify 20 of the remaining 25 samples. The phylogenetic tree constructed from the aligned amplicon and reference *atpA* sequences showed that the campylobacters in these 20 samples are very similar to “*Candidatus* Campylobacter infans” (Fig. 4A). Combining the *lpxA* and *atpA* results, 24 of 26 fecal samples were confirmed to contain “*Candidatus* Campylobacter infans,” and 19 of them were from exclusively breastfed infants, while 5 were from nonbreastfed infants (Fig. 4B), from five out of the seven GEMS countries, including The Gambia, Mali, Mozambique, India, and Pakistan.

As expected, genes encoding pathways for the utilization of amino acids and peptides, including amino acid transporters and peptidases, were identified in the “*Candidatus* Campylobacter infans” genome. The respiratory enzyme NADH:quinone oxidoreductase (also called complex I) is a major electron source in many bacteria, including C. jejuni (33), but all 14 *nuo* genes encoding complex I are absent in “*Candidatus* Campylobacter infans,” suggesting that it depends on other electron donors, such as hydrogen and formate, unless single-protein NADH dehydrogenases serve the same purpose. Genes required for utilizing hydrogen and formate as electron donors were identified in the metagenome, including those encoding the [Ni-Fe]-type hydrogenase HydABC, a nickel uptake system, and a formate dehydrogenase that can oxidize formate and release protons and electrons. Virulence genes, including those encoding invasion antigens (*ciaB* and *ciaD*), the zonula occludens toxin (*zot*), an outer membrane fibronectin-binding protein (*cadF*), heavy metal resistance genes, a multidrug efflux pump (*cmeABC*), and other drug resistance transporters, are potentially part of the “*Candidatus* Campylobacter infans” genome. Also, “*Candidatus* Campylobacter infans” may express an S-layer since we detected the presence of an S-layer secretion system (*sapDEF*), without homologs of the surface layer proteins (*sapA* and *sapB*), but other proteins may be involved.

### Prevalence and coinfection of *Campylobacter* spp. in GEMS.

As expected, since we used a subset of GEMS samples that tested positive for C. jejuni or C. coli, C. jejuni is the most prevalent species among symptomatic and asymptomatic *Campylobacter* infections, colonizing nearly twice as many infants as the total of all other species (107 colonized with C. jejuni and 68 colonized with other species). However, it is interesting to note that several other *Campylobacter* species are found, including C. coli (24 infants), “*Candidatus* Campylobacter infans” (24), and C. upsaliensis (18), and the order of abundance is generally consistent when split into the four groups (Fig. 4B). Among the 131 infants with *Campylobacter* infections, 36 (27.5%) were coinfected with different *Campylobacter* species, and among these, 8 (6.1%) were coinfected with three *Campylobacter* species (Table S4).

10.1128/mSphere.00735-19.9 TABLE S4 Coinfection with *Campylobacter* species in study subjects, detected by 16S rRNA sequencing and confirmed by *lpxA* multiplex PCR. Download Table S4, DOCX file, 0.01 MB. Copyright © 2020 Bian et al. 2020 Bian et al. This content is distributed under the terms of the Creative Commons Attribution 4.0 International license.

## DISCUSSION

In LMICs, diarrheal diseases are the second leading cause of mortality in children under 5 years of age, and infection with *Campylobacter* is one of the major causes of gastroenteritis in these children (1, 2, 7, 34). Previous studies suggest that breastfeeding protects infants from *Campylobacter-*induced diarrhea by reducing the ingestion of contaminated water/foods and lessening contact with unsanitary surroundings through closer interactions with the mother (7, 12, 28). In addition, breastfeeding increases exposure to fucosylated HMOs, particularly 2′-fucosyllactose in secretor mothers, which is predicted to act as a binding decoy to prevent C. jejuni colonization of the intestine (11). Yet exceedingly high rates of campylobacteriosis are reported among exclusively breastfed infants in LMICs (7). Consistent with these reports, our studies comparing 16S rRNA profiles of fecal DNA isolated from infants in GEMS show a trend of a higher *Campylobacter* abundance in exclusively breastfed than in nonbreastfed infants, leading us to evaluate the metabolic preferences among these isolates. We previously demonstrated that >50% of sequenced C. jejuni isolates possess the *fuc* locus for l-fucose catabolism (16), and the possession of this pathway provides a competitive colonization advantage in the piglet model of diarrheal disease (17). Since C. jejuni lacks fucosidases, it grows only on fucose-modified gut oligosaccharides in the presence of other gut microbes, such as Bacteroides vulgatus, suggesting a scavenging lifestyle within the intestine (35). Many commensal bacteria in the gut, including *Bifidobacterium* and *Bacteroides*, possess fucosidases and are predominant in breastfed infants (18–20), suggesting that breastfeeding may benefit *fuc*^+^ C. jejuni strains, particularly since fecal levels of free l-fucose can reach milligram amounts in breastfed infants (22).

In this study, we indeed found high levels of *Bifidobacterium* in breastfed infants, as expected. However, we found that although nonbreastfed infants were colonized with equal proportions of l-fucose-metabolizing and -nonmetabolizing C. jejuni and C. coli strains, the proportion of strains unable to metabolize l-fucose increased significantly in exclusively breastfed infants, although we cannot exclude the possibility that some infants were colonized with multiple strains of these species. This was particularly evident when comparing infants with symptomatic infections, who exhibited 6-fold-higher levels of this organism. Thus, HMOs may indeed function as decoys for *Campylobacter fuc*^+^ strains, and this may correlate with our recent observation that the *fuc* locus protein Cj0485 is necessary for *Campylobacter* l-fucose chemotaxis (16) and new studies in our laboratory comparing chemotaxis and sugar adhesion illustrating differences between strains capable of sugar metabolism and asaccharolytic strains (H. Nothaft, unpublished data). However, these results also suggest that there is a selection for C. jejuni or C. coli strains that are better capable of thriving within the infant gut. In addition to HMOs, breast milk contains high quantities of proteins (especially caseins) and their breakdown products (36), which could promote the growth of campylobacters, particularly since even carbohydrate-utilizing C. jejuni strains prefer amino acids such as serine, glutamate, and aspartate over fucose, indicating the existence of a metabolic hierarchy (35). Thus, a preference for the proteinaceous components in breast milk may make l-fucose or d-glucose utilization irrelevant. Additionally, the quantities of free l-fucose could be smaller than expected if mothers are not secretors or produce smaller quantities of HMOs due to nutrient availability (37, 38). Glutamine is one of the most abundant free amino acids in human milk, and the possession of the γ-glutamyl transpeptidase (GGT) enzyme has been negatively correlated with the presence of the fucose pathway in C. jejuni (29, 30), but only 15% of the isolated C. jejuni and C. coli strains lacking *fuc* examined in this study contain the *ggt* gene (lower than the 31% expected) (39), so it is likely that other carbon sources from breast milk fuel the metabolism of the isolated *Campylobacter* strains. In addition to C. jejuni and C. coli, “*Candidatus* Campylobacter infans” was also detected nearly three times as frequently in exclusively breastfed infants as in nonbreastfed infants, and the metagenomic data identified pathways for amino acid and peptide utilization, without any obvious clusters for carbohydrate metabolism. Based on the unexpected observation that exclusive breastfeeding increases the *Campylobacter* abundance in infants <1 year of age, more studies are needed to assess the effect of breast milk on *Campylobacter* infection, particularly since a recent study in preterm pigs fed human milk oligosaccharides showed increased levels of *Proteobacteria* dominated by *Campylobacter* and *Helicobacter* with longer HMO exposure (40). In particular, targeted epidemiological studies are required to determine whether higher *Campylobacter* levels are correlated with more severe dysenteric diarrhea. While C. jejuni or C. coli strains were isolated from all infants included in our study, 16S rRNA analysis did not detect *Campylobacter* in all the corresponding fecal DNA samples. This is consistent with our previous studies on chicken gut microbiome compositions that demonstrated that C. jejuni cannot be detected by 16S rRNA sequencing when <10^5^ CFU per g of cecal content are present (41, 42) and with studies by other groups that found that culture-based methods need to be used for bacteria present at levels below the detection threshold (<10^6^ CFU/g feces) to surpass the “depth bias” of genomic approaches (43, 44).

C. jejuni and C. coli are the two most commonly isolated pathogenic *Campylobacter* species worldwide (8); however, recently emerging non-C. jejuni/C. coli *Campylobacter* species have also been associated with gastrointestinal diseases and diarrhea (15). Here, we show that C. jejuni and C. coli are the predominant *Campylobacter* species in infants <1 year of age in GEMS, and non-C. jejuni/C. coli campylobacters, especially “*Candidatus* Campylobacter infans” and C. upsaliensis, are also prevalent in this age group. The prevalence of other *Campylobacter* species may be underestimated in this study since only fecal samples positive for C. jejuni and C. coli were included. Nonetheless, the new species “*Candidatus* Campylobacter infans” is detected at the same level as C. coli and accounts for approximately 1.6% of the fecal microbiome in exclusively breastfed infants with diarrhea and, in one extreme case, 83% of the total fecal microbiome, which suggests that “*Candidatus* Campylobacter infans” can be a relevant pathogen in infants. As the third most prevalent *Campylobacter* species in this study, C. upsaliensis has been previously reported to cause diarrhea in humans (45), and a recent study demonstrated that non-C. jejuni/C. coli species, such as C. hyointestinalis, account for more *Campylobacter* infections in children <2 years of age in Peru (15, 46). In the present study, coinfection with multiple *Campylobacter* species is not uncommon, which provides more opportunities for genomic rearrangements between campylobacters to escape selective pressures and alter virulence properties (47). Taken together, our data indicate that vaccines to prevent *Campylobacter* infection should not only focus on eliminating C. jejuni and C. coli but also consider other nonthermophilic species, including “*Candidatus* Campylobacter infans” and C. upsaliensis, for preventing diarrheal disease in infants in LMICs (42, 48). However, among all the infants with *Campylobacter* infection (both symptomatic and asymptomatic), approximately 16% are infected solely with *Campylobacter*, and 84% showed comorbidities with other diarrheal pathogens (viruses, other bacteria, and parasites), including 67% of infants diagnosed with *Campylobacter*-related diarrhea. Many studies have reported that coinfection with multiple intestinal pathogens is common in infants in LMICs, and one proteobacterial infection (such as Escherichia coli, *Salmonella*, and *Campylobacter*) increases the host’s susceptibility to other *Proteobacteria* (i.e., the proteobacterial bloom), so this should be kept in mind when considering the correlations observed in this study (1, 49, 50). Overall, our results indicate that approaches to control the risk of infection by other intestinal pathogens, such as sanitization, water treatment, and vaccination, are important to reduce diarrheal diseases in low-resource settings, but microbial gut compositions may also play a role.

In this study, we demonstrate that infants with diarrhea had the lowest fecal microbial diversity, consistent with data from previous studies that showed that diarrheal disease decreases the diversity of the gut microbiome (51). Diarrhea-free controls had significantly higher gut microbial diversity, which shows that asymptomatic *Campylobacter* infection does not devastate the gut microbiota as seen in diarrheal cases. When comparing cases and controls, the levels of several different genera were significantly elevated in controls among both exclusively breastfed and nonbreastfed infants, most of which were negatively correlated with *Campylobacter* yet positively correlated with each other, including *Blautia* and *Dorea*, *Blautia* and *Faecalibacterium*, and *Erysipelatoclostridium* and the Ruminococcus gnavus group. Among these genera, *Blautia* has been observed to have beneficial anti-inflammatory effects in illnesses such as graft-versus-host disease (52); however, other co-occurring bacteria (such as Faecalibacterium prausnitzii) may also protect against diarrhea (53), indicating that more studies need to be done to explore the potential protective roles of other microbes in diarrheal diseases.

Overall, our study points to the need to further explore the contributions of emerging pathogens such as “*Candidatus* Campylobacter infans” and C. upsaliensis to diarrheal disease in infants in LMICs and the potentially protective roles of intestinal bacteria, particularly those of the *Blautia* genus, that are associated with asymptomatic *Campylobacter* carriage. We also report the unexpected observation that a greater *Campylobacter* abundance is detected in exclusively breastfed infants with diarrheal disease, and further epidemiological studies with corroborating experimental work are warranted. These infants show a reduced incidence of infection with C. jejuni and C. coli fucose-utilizing strains, suggesting that HMOs may indeed function as decoys for *fuc*^+^ strains but also select for strains with metabolic properties that render them more capable of thriving in the infant gut. Current studies are focused on the interplay between C. jejuni chemotaxis, binding, and metabolism in exclusively breastfed infants and the development of inexpensive novel intervention and treatment strategies.

## MATERIALS AND METHODS

### Samples.

Fecal DNA samples were obtained from GEMS or the follow-up study GEMS1a, and the study design and methodology were previously described (24). Briefly, GEMS was a prospective case-control study of moderate-to-severe diarrhea (MSD) in children under 5 years of age conducted between 1 December 2007 and 3 March 2011 in seven low- and middle-income countries: The Gambia, Kenya, Mali, Mozambique, Bangladesh, India, and Pakistan. Cases with acute diarrhea were included, each representing a new episode and meeting at least one criterion of MSD (sunken eyes, loss of skin turgor, initiation of intravenous rehydration, dysentery, or hospitalization with diarrhea or dysentery). Controls had no diarrhea in 7 days and were included in the same demographic surveillance system area with cases and matched cases in age, sex, residence, and time. Illness management strategies, including feeding, fluid administration, and breastfeeding practices (no breastfeeding, partly breastfeeding, and exclusively breastfeeding), were queried from the caretakers. A follow-up study called GEMS1a also enrolled children with MSD as well as children with less-severe diarrhea (LSD) (acute diarrhea without signs of MSD) and matched controls from 31 October 2011 to 14 November 2012, using essentially the same methodology as the one for GEMS. To study the spread of *Campylobacter* and the effect of breastfeeding on campylobacteriosis, we included DNA samples isolated from stools of cases (both MSD and LSD) and controls under 1 year of age, whose caretakers indicated that they were exclusively breastfed or not breastfed (infants who were still breastfed but also received complementary food were excluded from the analysis), with *Campylobacter* isolated from the fecal samples using selective Campy-BAP plates for cephalothin-resistant *Campylobacter* species, including most C. jejuni and C. coli strains, grown at 42°C for 3 days. Subsequently, one single colony per sample was subcultured and used in this study (9).

### 16S rRNA sequencing and data analysis.

The V6-V8 and V4 hypervariable regions of the 16S rRNA gene were amplified from fecal DNA using universal primers described previously (42, 54). The amplification products were cleaned up, normalized, barcoded, and sent to the Georgia Genomics and Bioinformatics Core (GGBC) for sequencing using the Illumina MiSeq PE300 kit. Normalization was based on the double-stranded DNA (dsDNA) concentration detected by using the NanoDrop One^C^ system (Thermo Scientific) and a Qubit 2.0 fluorometer (Thermo Scientific). A total of 12,387,812 sequences were obtained, with a median of 53,166 sequences per sample. DADA2, an open-source software package for modeling and correcting Illumina-sequenced-amplicon errors, was used in QIIME2 to filter, trim, dereplicate, and join the yielded paired-end sequences and construct an amplicon sequence variant (ASV) table for further analysis (55–57). The alpha diversity of the fecal microbiome was calculated using the Shannon index in QIIME2. Vsearch was used to cluster representative sequences against the SILVA SSU r128 database with 99% identity (58) to known operational taxonomic units (OTUs). Correlation between microbial OTUs was calculated using SparCC, a method to estimate correlation values from compositional data (59).

### Fucose permease gene (*fucP*) and γ-glutamyl transpeptidase gene (*ggt*) screening and l-fucose/d-glucose utilization experiments with C. jejuni and C. coli strains.

To identify the potential fucose utilization ability of *Campylobacter* strains isolated in GEMS, we screened for the *fucP* gene in all GEMS-isolated *Campylobacter* strains (C. jejuni and C. coli) by colony or genomic DNA PCR using primers designed to amplify the *fucP* gene of C. jejuni NCTC 11168 (fucP-F/R [CAT GAA AGT GGC TTT TTA CAG/ATT TTT TTC ATC ACC AAG CTT TG]) in a multiplex format with 16S rRNA control primers (16S-F/R [AAT GGC TTA ACC ATT AAA CTG C/AAC TAA ATA CGT GGG TTG CG]), which was confirmed to also work for C. coli strains (data not shown). To verify that a *fucP* PCR product corresponded to a strain’s ability to use fucose, we performed growth experiments in a subset of the strains lacking *fucP* and all *fucP*^+^ strains as described previously (16). Briefly, *Campylobacter* strains were adjusted to an optical density at 600 nm (OD_600_) of 0.05 and inoculated into 5 ml MEMα (minimum essential medium α; Thermo Fisher Scientific) (with 20 μM iron added) with or without 25 mM l-fucose. The inoculated culture was incubated microaerobically at 37°C for 18 h, and the OD_600_ was measured using the NanoDrop One^C^ system (Thermo Scientific). Screening for the *ggt* gene was carried out as previously described (29). To test for d-glucose utilization, we performed growth experiments as described above, using a random subset of 33 strains in DMEM (Dulbecco’s modified Eagle’s medium) without glucose (Thermo Fisher Scientific), and the strains were incubated microaerobically for 48 h. Campylobacter cuniculorum LMG 24588 was used as the positive control.

### Verification of C. jejuni, C. coli, C. upsaliensis, C. lari, and C. hyointestinalis.

Representative sequences of 16S rRNA (V6-V8) of *Campylobacter* classified by Scikit-learn (60) in QIIME2 were aligned and classified using SINA with the SILVA SSU r128 database, and bacterial species with a 0.99 minimum identity to the query sequence were obtained (61). A multiplex PCR of the lipid A biosynthesis gene *lpxA* was used to confirm the presence of C. jejuni, C. coli, C. upsaliensis, and C. lari in fecal DNA, as previously described (31). A novel *lpxA* forward primer was designed here for C. hyointestinalis (lpxAC.hyo [CAA AGA CGC GGT TTT GGG CGA TGA AGT CGT GG]). This primer is compatible with the *lpxA* primers described previously (31), yielding (in conjunction with the previously described reverse primer) a product of 285 bp. The lpxAC.hyo and lpxARKK2m primer set was used to verify the presence of C. hyointestinalis (both subspecies) in fecal DNA.

### Detection of a new *Campylobacter* species.

Illumina reads were initially trimmed to remove adapters and quality filtered (>Q20) using Trimmomatic (v0.39) (62), and human genome contamination was then removed by mapping reads to the human GRCh37/hg19 reference genome with Bowtie2 (v2.1.0) (63). Next, filtered reads were submitted to MG-RAST (64) for gene function prediction and taxonomic abundance determination, while the reads were assembled and the taxonomic abundance was confirmed using the following programs: metaSPAdes (assembly; v3.12.0) (65), Kraken2 (taxonomic abundance; v2.0.8) (66), and Bracken (taxonomic abundance; v2.0.0) (67). The metaSPAdes assembly resulted in 33 contigs identified as *Campylobacter*, including 24 contigs of >5,000 bp, while an additional assembly with Newbler (v2.6) yielded 75 contigs identified as *Campylobacter*, including 56 contigs of >5,000 bp. Both sets of assembled *Campylobacter* contigs from metaSPAdes and Newbler were independently analyzed for completeness and contamination using CheckM software (v1.0.12) (68), which demonstrated that the 33 contigs from metaSPAdes were 94.2% complete, with 4.9% contamination, whereas the 75 contigs from Newbler were 98.5% complete, with only 0.19% contamination (classified as an extremely low level of contamination and within the margin of error for the CheckM software). Therefore, all additional metagenomic *Campylobacter* analyses were conducted with the contigs from the Newbler assembly. Coding sequences present within the 75-contig set were determined using GeneMark (69). A custom protein database was constructed, which combined the proteomes derived from all currently validated (and fully annotated) *Campylobacter* and *Arcobacter* genomes (one to six proteomes per taxon) present in GenBank. The metagenomic proteome was compared to this custom database through an all-versus-all pairwise BLASTP analysis. Genes within the metagenome were assigned a function based on matches above the minimum requirement of 40% similarity and where the match length was ≥75% of the subject and query protein lengths. The sequences of 20 core genes (*aroC*, *atpA*, *dnaN*, *eno*, *fabH*, *frr*, *glnA*, *groEL*, *hemB*, *ileS*, *lpxA*, *miaB*, *mrp*, *nrdB*, *pnp*, *prfA*, *queA*, *speA*, *spoT*, and *tkt*) and their cognate proteins were extracted from the metagenome and 50 *Campylobacter*, *Arcobacter*, and *Sulfurospirillum* genomes. These gene and protein sets were concatenated in the order mentioned above for each genome and aligned using CLUSTALX. Neighbor-joining phylogenetic trees were constructed using MEGA v6 (70): the nucleotide tree was constructed using the Kimura two-parameter model, and the amino acid tree was constructed using the Poisson model. Additionally, the 75 *Campylobacter* contigs were concatenated and used for an average nucleotide identity (ANI) analysis (71), along with the genomes of 45 *Campylobacter*, *Arcobacter*, *Sulfurimonas*, *Sulfurospirillum*, and *Helicobacter* taxa. Using the metagenome sequence, novel *lpxA* (lpxAC.GEMS [GCA AAA AGC AGT GGC GAG GGT TG], compatible with lpxARKK2m) and *atpA* (atpA-F/R [GTG AGC GCA AAA TTA AAA GCA G/TTA TTC AGC ACT AAA AGT AGC C]) primers were designed and used to verify the presence of this putative new *Campylobacter* species in the other 25 fecal samples. The *atpA* sequences were aligned using CLUSTALW, and neighbor-joining phylogenetic trees were constructed with MEGA v7 using the Tamura three-parameter method (72).

### Statistics.

For analysis of the fecal microbiome, a Kruskal-Wallis test was used to compare alpha diversity values between different groups; differences in the relative abundances of bacterial phyla between groups were calculated by a Kruskal-Wallis H test and corrected by the false discovery rate (FDR) in STAMP (73). For analysis of strains capable of metabolizing fucose, a chi-square test was used to determine if the proportion of samples that can metabolize fucose in both cases and controls significantly differed from 50%.

### Data availability.

The metagenome containing “*Candidatus* Campylobacter infans” can be found in the NCBI database (accession number SPMW00000000). The 16S rRNA sequencing data can also be found in the NCBI database under the following accession numbers: V4 data (PRJNA596502); V6-V8, samples 1-100, data (PRJNA596501); V6-V8, samples 101-233, data (PRJNA596500).

## References

[1] Lozano R, Naghavi M, Foreman K, Lim S, Shibuya K, Aboyans V, Abraham J, Adair T, Aggarwal R, Ahn SY, Alvarado M, Anderson HR, Anderson LM, Andrews KG, Atkinson C, Baddour LM, Barker-Collo S, Bartels DH, Bell ML, Benjamin EJ, Bennett D, Bhalla K, Bikbov B, Bin Abdulhak A, Birbeck G, Blyth F, Bolliger I, Boufous S, Bucello C, Burch M, Burney P, Carapetis J, Chen H, Chou D, Chugh SS, Coffeng LE, Colan SD, Colquhoun S, Colson KE, Condon J, Connor MD, Cooper LT, Corriere M, Cortinovis M, de Vaccaro KC, Couser W, Cowie BC, Criqui MH, Cross M, 2012 Global and regional mortality from 235 causes of death for 20 age groups in 1990 and 2010: a systematic analysis for the Global Burden of Disease Study 2010. Lancet 380:2095–2128. doi:10.1016/S0140-6736(12)61728-0. 23245604 PMC10790329

[2] Liu J, Platts-Mills JA, Juma J, Kabir F, Nkeze J, Okoi C, Operario DJ, Uddin J, Ahmed S, Alonso PL, Antonio M, Becker SM, Blackwelder WC, Breiman RF, Faruque ASG, Fields B, Gratz J, Haque R, Hossain A, Hossain MJ, Jarju S, Qamar F, Iqbal NT, Kwambana B, Mandomando I, McMurry TL, Ochieng C, Ochieng JB, Ochieng M, Onyango C, Panchalingam S, Kalam A, Aziz F, Qureshi S, Ramamurthy T, Roberts JH, Saha D, Sow SO, Stroup SE, Sur D, Tamboura B, Taniuchi M, Tennant SM, Toema D, Wu Y, Zaidi A, Nataro JP, Kotloff KL, Levine MM, Houpt ER 2016 Use of quantitative molecular diagnostic methods to identify causes of diarrhoea in children: a reanalysis of the GEMS case-control study. Lancet 388:1291–1301. doi:10.1016/S0140-6736(16)31529-X. 27673470 PMC5471845

[3] Coker AO, Isokpehi RD, Thomas BN, Amisu KO, Obi CL 2002 Human campylobacteriosis in developing countries. Emerg Infect Dis 8:237–243. doi:10.3201/eid0803.010233. 11927019 PMC2732465

[4] Chen ML, Ge Z, Fox JG, Schauer DB 2006 Disruption of tight junctions and induction of proinflammatory cytokine responses in colonic epithelial cells by *Campylobacter jejuni*. Infect Immun 74:6581–6589. doi:10.1128/IAI.00958-06. 17015453 PMC1698078

[5] Kalischuk LD, Leggett F, Inglis GD 2010 *Campylobacter jejuni* induces transcytosis of commensal bacteria across the intestinal epithelium through M-like cells. Gut Pathog 2:14. doi:10.1186/1757-4749-2-14. 21040540 PMC2987776

[6] Lee G, Pan W, Penataro Yori P, Paredes Olortegui M, Tilley D, Gregory M, Oberhelman R, Burga R, Chavez CB, Kosek M 2013 Symptomatic and asymptomatic *Campylobacter* infections associated with reduced growth in Peruvian children. PLoS Negl Trop Dis 7:e2036. doi:10.1371/journal.pntd.0002036. 23383356 PMC3561130

[7] Amour C, Gratz J, Mduma E, Svensen E, Rogawski ET, McGrath M, Seidman JC, McCormick BJ, Shrestha S, Samie A, Mahfuz M, Qureshi S, Hotwani A, Babji S, Trigoso DR, Lima AA, Bodhidatta L, Bessong P, Ahmed T, Shakoor S, Kang G, Kosek M, Guerrant RL, Lang D, Gottlieb M, Houpt ER, Platts-Mills JA, for the Etiology, Risk Factors, and Interactions of Enteric Infections and Malnutrition and the Consequences for Child Health and Development Project (MAL-ED) Network Investigators. 2016 Epidemiology and impact of *Campylobacter* infection in children in 8 low-resource settings: results from the MAL-ED study. Clin Infect Dis 63:1171–1179. doi:10.1093/cid/ciw542. 27501842 PMC5064165

[8] Kaakoush NO, Castano-Rodriguez N, Mitchell HM, Man SM 2015 Global epidemiology of *Campylobacter* infection. Clin Microbiol Rev 28:687–720. doi:10.1128/CMR.00006-15. 26062576 PMC4462680

[9] Jacob P, Mdegela RH, Nonga HE 2011 Comparison of Cape Town and Skirrow’s *Campylobacter* isolation protocols in humans and broilers in Morogoro, Tanzania. Trop Anim Health Prod 43:1007–1013. doi:10.1007/s11250-011-9799-z. 21359592

[10] Le Doare K, Holder B, Bassett A, Pannaraj PS 2018 Mother’s milk: a purposeful contribution to the development of the infant microbiota and immunity. Front Immunol 9:361. doi:10.3389/fimmu.2018.00361. 29599768 PMC5863526

[11] Ruiz-Palacios GM, Cervantes LE, Ramos P, Chavez-Munguia B, Newburg DS 2003 *Campylobacter jejuni* binds intestinal H(O) antigen (Fuca1, 2Galb1, 4GlcNAc), and fucosyloligosaccharides of human milk inhibit its binding and infection. J Biol Chem 278:14112–14120. doi:10.1074/jbc.M207744200. 12562767

[12] Ruiz-Palacios GM, Calva JJ, Pickering LK, Lopez-Vidal Y, Volkow P, Pezzarossi H, West MS 1990 Protection of breast-fed infants against *Campylobacter* diarrhea by antibodies in human milk. J Pediatr 116:707–713. doi:10.1016/s0022-3476(05)82652-6. 2329419

[13] Mohan V 2015 The role of probiotics in the inhibition of *Campylobacter jejuni* colonization and virulence attenuation. Eur J Clin Microbiol Infect Dis 34:1503–1513. doi:10.1007/s10096-015-2392-z. 25934376

[14] Ho NT, Li F, Lee-Sarwar KA, Tun HM, Brown BP, Pannaraj PS, Bender JM, Azad MB, Thompson AL, Weiss ST, Azcarate-Peril MA, Litonjua AA, Kozyrskyj AL, Jaspan HB, Aldrovandi GM, Kuhn L 2018 Meta-analysis of effects of exclusive breastfeeding on infant gut microbiota across populations. Nat Commun 9:4169. doi:10.1038/s41467-018-06473-x. 30301893 PMC6177445

[15] Francois R, Yori PP, Rouhani S, Siguas Salas M, Paredes Olortegui M, Rengifo Trigoso D, Pisanic N, Burga R, Meza R, Meza Sanchez G, Gregory MJ, Houpt ER, Platts-Mills JA, Kosek MN 2018 The other *Campylobacters*: not innocent bystanders in endemic diarrhea and dysentery in children in low-income settings. PLoS Negl Trop Dis 12:e0006200. doi:10.1371/journal.pntd.0006200. 29415075 PMC5819825

[16] Dwivedi R, Nothaft H, Garber J, Xin Kin L, Stahl M, Flint A, van Vliet AH, Stintzi A, Szymanski CM 2016 L-Fucose influences chemotaxis and biofilm formation in *Campylobacter jejuni*. Mol Microbiol 101:575–589. doi:10.1111/mmi.13409. 27145048

[17] Stahl M, Friis LM, Nothaft H, Liu X, Li J, Szymanski CM, Stintzi A 2011 L-Fucose utilization provides *Campylobacter jejuni* with a competitive advantage. Proc Natl Acad Sci U S A 108:7194–7199. doi:10.1073/pnas.1014125108. 21482772 PMC3084102

[18] Marcobal A, Barboza M, Sonnenburg ED, Pudlo N, Martens EC, Desai P, Lebrilla CB, Weimer BC, Mills DA, German JB, Sonnenburg JL 2011 *Bacteroides* in the infant gut consume milk oligosaccharides via mucus-utilization pathways. Cell Host Microbe 10:507–514. doi:10.1016/j.chom.2011.10.007. 22036470 PMC3227561

[19] Bunesova V, Lacroix C, Schwab C 2016 Fucosyllactose and L-fucose utilization of infant *Bifidobacterium longum* and *Bifidobacterium kashiwanohense*. BMC Microbiol 16:248. doi:10.1186/s12866-016-0867-4. 27782805 PMC5080750

[20] Matsuki T, Yahagi K, Mori H, Matsumoto H, Hara T, Tajima S, Ogawa E, Kodama H, Yamamoto K, Yamada T, Matsumoto S, Kurokawa K 2016 A key genetic factor for fucosyllactose utilization affects infant gut microbiota development. Nat Commun 7:11939. doi:10.1038/ncomms11939. 27340092 PMC4931012

[21] Hofreuter D 2014 Defining the metabolic requirements for the growth and colonization capacity of *Campylobacter jejuni*. Front Cell Infect Microbiol 4:137. doi:10.3389/fcimb.2014.00137. 25325018 PMC4178425

[22] Xu G, Amicucci MJ, Cheng Z, Galermo AG, Lebrilla CB 2017 Revisiting monosaccharide analysis—quantitation of a comprehensive set of monosaccharides using dynamic multiple reaction monitoring. Analyst 143:200–207. doi:10.1039/c7an01530e. 29186215 PMC6203862

[23] Vegge CS, Jansen van Rensburg MJ, Rasmussen JJ, Maiden MC, Johnsen LG, Danielsen M, MacIntyre S, Ingmer H, Kelly DJ 2016 Glucose metabolism via the Entner-Doudoroff pathway in *Campylobacter*: a rare trait that enhances survival and promotes biofilm formation in some isolates. Front Microbiol 7:1877. doi:10.3389/fmicb.2016.01877. 27920773 PMC5118423

[24] Kotloff KL, Blackwelder WC, Nasrin D, Nataro JP, Farag TH, van Eijk A, Adegbola RA, Alonso PL, Breiman RF, Faruque AS, Saha D, Sow SO, Sur D, Zaidi AK, Biswas K, Panchalingam S, Clemens JD, Cohen D, Glass RI, Mintz ED, Sommerfelt H, Levine MM 2012 The Global Enteric Multicenter Study (GEMS) of diarrheal disease in infants and young children in developing countries: epidemiologic and clinical methods of the case/control study. Clin Infect Dis 55(Suppl 4):S232–S245. doi:10.1093/cid/cis753. 23169936 PMC3502307

[25] Panchalingam S, Antonio M, Hossain A, Mandomando I, Ochieng B, Oundo J, Ramamurthy T, Tamboura B, Zaidi AK, Petri W, Houpt E, Murray P, Prado V, Vidal R, Steele D, Strockbine N, Sansonetti P, Glass RI, Robins-Browne RM, Tauschek M, Svennerholm AM, Berkeley LY, Kotloff K, Levine MM, Nataro JP 2012 Diagnostic microbiologic methods in the GEMS-1 case/control study. Clin Infect Dis 55(Suppl 4):S294–S302. doi:10.1093/cid/cis754. 23169941 PMC3502308

[26] Gomez DE, Arroyo LG, Costa MC, Viel L, Weese JS 2017 Characterization of the fecal bacterial microbiota of healthy and diarrheic dairy calves. J Vet Intern Med 31:928–939. doi:10.1111/jvim.14695. 28390070 PMC5435056

[27] Graspeuntner S, Loeper N, Kunzel S, Baines JF, Rupp J 2018 Selection of validated hypervariable regions is crucial in 16S-based microbiota studies of the female genital tract. Sci Rep 8:9678. doi:10.1038/s41598-018-27757-8. 29946153 PMC6018735

[28] Yu ZT, Nanthakumar NN, Newburg DS 2016 The human milk oligosaccharide 2′-fucosyllactose quenches *Campylobacter jejuni*-induced inflammation in human epithelial cells HEp-2 and HT-29 and in mouse intestinal mucosa. J Nutr 146:1980–1990. doi:10.3945/jn.116.230706. 27629573 PMC5037868

[29] Muraoka WT, Zhang Q 2011 Phenotypic and genotypic evidence for L-fucose utilization by *Campylobacter jejuni*. J Bacteriol 193:1065–1075. doi:10.1128/JB.01252-10. 21193610 PMC3067607

[30] Zhang Z, Adelman AS, Rai D, Boettcher J, Lőnnerdal B 2013 Amino acid profiles in term and preterm human milk through lactation: a systematic review. Nutrients 5:4800–4821. doi:10.3390/nu5124800. 24288022 PMC3875913

[31] Klena JD, Parker CT, Knibb K, Ibbitt JC, Devane PM, Horn ST, Miller WG, Konkel ME 2004 Differentiation of *Campylobacter coli*, *Campylobacter jejuni*, *Campylobacter lari*, and *Campylobacter upsaliensis* by a multiplex PCR developed from the nucleotide sequence of the lipid A gene *lpxA*. J Clin Microbiol 42:5549–5557. doi:10.1128/JCM.42.12.5549-5557.2004. 15583280 PMC535264

[32] Bian X, Huynh S, Chapman MH, Szymanski CM, Parker CT, Miller WG 2018 Draft genome sequences of nine *Campylobacter hyointestinalis* subsp. *lawsonii* strains. Microbiol Resour Announc 7:e01016-18. doi:10.1128/MRA.01016-18. 30533618 PMC6256594

[33] Weerakoon DR, Olson JW 2008 The *Campylobacter jejuni* NADH:ubiquinone oxidoreductase (complex I) utilizes flavodoxin rather than NADH. J Bacteriol 190:915–925. doi:10.1128/JB.01647-07. 18065531 PMC2223568

[34] Centers for Disease Control and Prevention. 2015 Global diarrhea burden: global water, sanitation and hygiene. Centers for Disease Control and Prevention, Atlanta, GA https://www.cdc.gov/healthywater/global/diarrhea-burden.html.

[35] Garber JM, Nothaft H, Pluvinage B, Stahl M, Bian X, Enriquez A, Butcher J, Huang H, Line E, Gerlt JA, Glushka J, Stintzi A, Boraston AB, Szymanski CM No longer asaccharolytic metabolism in *Campylobacter jejuni*. Commun Biol, in press. 10.1038/s42003-019-0727-5 PMC6946681 31925306

[36] Ballard O, Morrow AL 2013 Human milk composition: nutrients and bioactive factors. Pediatr Clin North Am 60:49–74. doi:10.1016/j.pcl.2012.10.002. 23178060 PMC3586783

[37] Castanys-Munoz E, Martin MJ, Prieto PA 2013 2′-Fucosyllactose: an abundant, genetically determined soluble glycan present in human milk. Nutr Rev 71:773–789. doi:10.1111/nure.12079. 24246032

[38] Smilowitz JT, O’Sullivan A, Barile D, German JB, Lönnerdal B, Slupsky CM 2013 The human milk metabolome reveals diverse oligosaccharide profiles. J Nutr 143:1709–1718. doi:10.3945/jn.113.178772. 24027187 PMC4083237

[39] de Haan CPA, Llarena A-K, Revez J, Hänninen M-L 2012 Association of *Campylobacter jejuni* metabolic traits with multilocus sequence types. Appl Environ Microbiol 78:5550–5554. doi:10.1128/AEM.01023-12. 22660710 PMC3406166

[40] Rudloff S, Kuntz S, Ostenfeldt Rasmussen S, Roggenbuck M, Sprenger N, Kunz C, Sangild PT, Brandt Bering S 2019 Metabolism of milk oligosaccharides in preterm pigs sensitive to necrotizing enterocolitis. Front Nutr 6:23. doi:10.3389/fnut.2019.00023. 30931310 PMC6424005

[41] Nothaft H, Perez-Muñoz ME, Gouveia GJ, Duar RM, Wanford JJ, Lango-Scholey L, Panagos CG, Srithayakumar V, Plastow GS, Coros C, Bayliss CD, Edison AS, Walter J, Szymanski CM 2017 Coadministration of the *Campylobacter jejuni* N-glycan-based vaccine with probiotics improves vaccine performance in broiler chickens. Appl Environ Microbiol 83:e01523-17. doi:10.1128/AEM.01523-17. 28939610 PMC5691412

[42] Nothaft H, Davis B, Lock YY, Perez-Munoz ME, Vinogradov E, Walter J, Coros C, Szymanski CM 2016 Engineering the *Campylobacter jejuni* N-glycan to create an effective chicken vaccine. Sci Rep 6:26511. doi:10.1038/srep26511. 27221144 PMC4879521

[43] Lagier JC, Armougom F, Million M, Hugon P, Pagnier I, Robert C, Bittar F, Fournous G, Gimenez G, Maraninchi M, Trape JF, Koonin EV, La Scola B, Raoult D 2012 Microbial culturomics: paradigm shift in the human gut microbiome study. Clin Microbiol Infect 18:1185–1193. doi:10.1111/1469-0691.12023. 23033984

[44] Lau JT, Whelan FJ, Herath I, Lee CH, Collins SM, Bercik P, Surette MG 2016 Capturing the diversity of the human gut microbiota through culture-enriched molecular profiling. Genome Med 8:72. doi:10.1186/s13073-016-0327-7. 27363992 PMC4929786

[45] Couturier BA, Hale DC, Couturier MR 2012 Association of *Campylobacter upsaliensis* with persistent bloody diarrhea. J Clin Microbiol 50:3792–3794. doi:10.1128/JCM.01807-12. 22915607 PMC3486253

[46] Platts-Mills JA, Liu J, Gratz J, Mduma E, Amour C, Swai N, Taniuchi M, Begum S, Yori P, Tilley DH, Lee G, Shen Z, Whary MT, Fox JG, McGrath M, Kosek M, Haque R, Houpt ER 2014 Detection of *Campylobacter* in stool and determination of significance by culture, enzyme immunoassay, and PCR in developing countries. J Clin Microbiol 52:1074–1080. doi:10.1128/JCM.02935-13. 24452175 PMC3993515

[47] Burnham PM, Hendrixson DR 2018 *Campylobacter jejuni*: collective components promoting a successful enteric lifestyle. Nat Rev Microbiol 16:551–565. doi:10.1038/s41579-018-0037-9. 29892020

[48] Monteiro MA, Baqar S, Hall ER, Chen YH, Porter CK, Bentzel DE, Applebee L, Guerry P 2009 Capsule polysaccharide conjugate vaccine against diarrheal disease caused by *Campylobacter jejuni*. Infect Immun 77:1128–1136. doi:10.1128/IAI.01056-08. 19114545 PMC2643618

[49] Moyo SJ, Kommedal O, Blomberg B, Hanevik K, Tellevik MG, Maselle SY, Langeland N 2017 Comprehensive analysis of prevalence, epidemiologic characteristics, and clinical characteristics of monoinfection and coinfection in diarrheal diseases in children in Tanzania. Am J Epidemiol 186:1074–1083. doi:10.1093/aje/kwx173. 28541454 PMC5860328

[50] Winter SE, Baumler AJ 2014 Why related bacterial species bloom simultaneously in the gut: principles underlying the ‘like will to like’ concept. Cell Microbiol 16:179–184. doi:10.1111/cmi.12245. 24286560 PMC4013256

[51] Chang JY, Antonopoulos DA, Kalra A, Tonelli A, Khalife WT, Schmidt TM, Young VB 2008 Decreased diversity of the fecal microbiome in recurrent *Clostridium difficile*-associated diarrhea. J Infect Dis 197:435–438. doi:10.1086/525047. 18199029

[52] Jenq RR, Taur Y, Devlin SM, Ponce DM, Goldberg JD, Ahr KF, Littmann ER, Ling L, Gobourne AC, Miller LC, Docampo MD, Peled JU, Arpaia N, Cross JR, Peets TK, Lumish MA, Shono Y, Dudakov JA, Poeck H, Hanash AM, Barker JN, Perales MA, Giralt SA, Pamer EG, van den Brink MR 2015 Intestinal *Blautia* is associated with reduced death from graft-versus-host disease. Biol Blood Marrow Transplant 21:1373–1383. doi:10.1016/j.bbmt.2015.04.016. 25977230 PMC4516127

[53] Quevrain E, Maubert MA, Michon C, Chain F, Marquant R, Tailhades J, Miquel S, Carlier L, Bermudez-Humaran LG, Pigneur B, Lequin O, Kharrat P, Thomas G, Rainteau D, Aubry C, Breyner N, Afonso C, Lavielle S, Grill JP, Chassaing G, Chatel JM, Trugnan G, Xavier R, Langella P, Sokol H, Seksik P 2016 Identification of an anti-inflammatory protein from *Faecalibacterium prausnitzii*, a commensal bacterium deficient in Crohn’s disease. Gut 65:415–425. doi:10.1136/gutjnl-2014-307649. 26045134 PMC5136800

[54] Caporaso JG, Lauber CL, Walters WA, Berg-Lyons D, Huntley J, Fierer N, Owens SM, Betley J, Fraser L, Bauer M, Gormley N, Gilbert JA, Smith G, Knight R 2012 Ultra-high-throughput microbial community analysis on the Illumina HiSeq and MiSeq platforms. ISME J 6:1621–1624. doi:10.1038/ismej.2012.8. 22402401 PMC3400413

[55] Callahan BJ, McMurdie PJ, Rosen MJ, Han AW, Johnson AJ, Holmes SP 2016 DADA2: high-resolution sample inference from Illumina amplicon data. Nat Methods 13:581–583. doi:10.1038/nmeth.3869. 27214047 PMC4927377

[56] Bokulich NA, Kaehler BD, Rideout JR, Dillon M, Bolyen E, Knight R, Huttley GA, Caporaso JG 2018 Optimizing taxonomic classification of marker-gene amplicon sequences with QIIME 2’s q2-feature-classifier plugin. Microbiome 6:90. doi:10.1186/s40168-018-0470-z. 29773078 PMC5956843

[57] Caporaso JG, Kuczynski J, Stombaugh J, Bittinger K, Bushman FD, Costello EK, Fierer N, Pena AG, Goodrich JK, Gordon JI, Huttley GA, Kelley ST, Knights D, Koenig JE, Ley RE, Lozupone CA, McDonald D, Muegge BD, Pirrung M, Reeder J, Sevinsky JR, Turnbaugh PJ, Walters WA, Widmann J, Yatsunenko T, Zaneveld J, Knight R 2010 QIIME allows analysis of high-throughput community sequencing data. Nat Methods 7:335–336. doi:10.1038/nmeth.f.303. 20383131 PMC3156573

[58] Rognes T, Flouri T, Nichols B, Quince C, Mahe F 2016 VSEARCH: a versatile open source tool for metagenomics. PeerJ 4:e2584. doi:10.7717/peerj.2584. 27781170 PMC5075697

[59] Friedman J, Alm EJ 2012 Inferring correlation networks from genomic survey data. PLoS Comput Biol 8:e1002687. doi:10.1371/journal.pcbi.1002687. 23028285 PMC3447976

[60] Pedregosa F, Varoquaux G, Gramfort A, Michel V, Thirion B, Grisel O, Blondel M, Prettenhofer P, Weiss R, Dubourg V, Vanderplas J 2011 Scikit-learn: machine learning in Python. J Mach Learn Res 12:2825–2830.

[61] Quast C, Pruesse E, Yilmaz P, Gerken J, Schweer T, Yarza P, Peplies J, Glockner FO 2013 The SILVA ribosomal RNA gene database project: improved data processing and Web-based tools. Nucleic Acids Res 41:D590–D596. doi:10.1093/nar/gks1219. 23193283 PMC3531112

[62] Bolger AM, Lohse M, Usadel B 2014 Trimmomatic: a flexible trimmer for Illumina sequence data. Bioinformatics 30:2114–2120. doi:10.1093/bioinformatics/btu170. 24695404 PMC4103590

[63] Langmead B, Salzberg SL 2012 Fast gapped-read alignment with Bowtie 2. Nat Methods 9:357–359. doi:10.1038/nmeth.1923. 22388286 PMC3322381

[64] Wilke A, Bischof J, Gerlach W, Glass E, Harrison T, Keegan KP, Paczian T, Trimble WL, Bagchi S, Grama A, Chaterji S, Meyer F 2016 The MG-RAST metagenomics database and portal in 2015. Nucleic Acids Res 44:D590–D594. doi:10.1093/nar/gkv1322. 26656948 PMC4702923

[65] Nurk S, Meleshko D, Korobeynikov A, Pevzner PA 2017 metaSPAdes: a new versatile metagenomic assembler. Genome Res 27:824–834. doi:10.1101/gr.213959.116. 28298430 PMC5411777

[66] Wood DE, Salzberg SL 2014 Kraken: ultrafast metagenomic sequence classification using exact alignments. Genome Biol 15:R46. doi:10.1186/gb-2014-15-3-r46. 24580807 PMC4053813

[67] Lu J, Breitwieser FP, Thielen P, Salzberg SL 2017 Bracken: estimating species abundance in metagenomics data. PeerJ Comput Sci 3:e104. doi:10.7717/peerj-cs.104. PMC12016282 40271438

[68] Parks DH, Imelfort M, Skennerton CT, Hugenholtz P, Tyson GW 2015 CheckM: assessing the quality of microbial genomes recovered from isolates, single cells, and metagenomes. Genome Res 25:1043–1055. doi:10.1101/gr.186072.114. 25977477 PMC4484387

[69] Besemer J, Borodovsky M 2005 GeneMark: Web software for gene finding in prokaryotes, eukaryotes and viruses. Nucleic Acids Res 33:W451–W454. doi:10.1093/nar/gki487. 15980510 PMC1160247

[70] Tamura K, Stecher G, Peterson D, Filipski A, Kumar S 2013 MEGA6: Molecular Evolutionary Genetics Analysis version 6.0. Mol Biol Evol 30:2725–2729. doi:10.1093/molbev/mst197. 24132122 PMC3840312

[71] Richter M, Rosselló-Móra R 2009 Shifting the genomic gold standard for the prokaryotic species definition. Proc Natl Acad Sci U S A 106:19126–19131. doi:10.1073/pnas.0906412106. 19855009 PMC2776425

[72] Kumar S, Stecher G, Tamura K 2016 MEGA7: Molecular Evolutionary Genetics Analysis version 7.0 for bigger datasets. Mol Biol Evol 33:1870–1874. doi:10.1093/molbev/msw054. 27004904 PMC8210823

[73] Parks DH, Tyson GW, Hugenholtz P, Beiko RG 2014 STAMP: statistical analysis of taxonomic and functional profiles. Bioinformatics 30:3123–3124. doi:10.1093/bioinformatics/btu494. 25061070 PMC4609014

