# Supplementary figures and images for: Campylobacter Abundance in Breastfed Infants and Identification of a New Species in the Global Enterics Multicenter Study
Source: mSphere. 2020 Jan 15;5(1):e00735-19. doi: 10.1128/mSphere.00735-19 (PMC6968651; doi:10.1128/mSphere.00735-19)

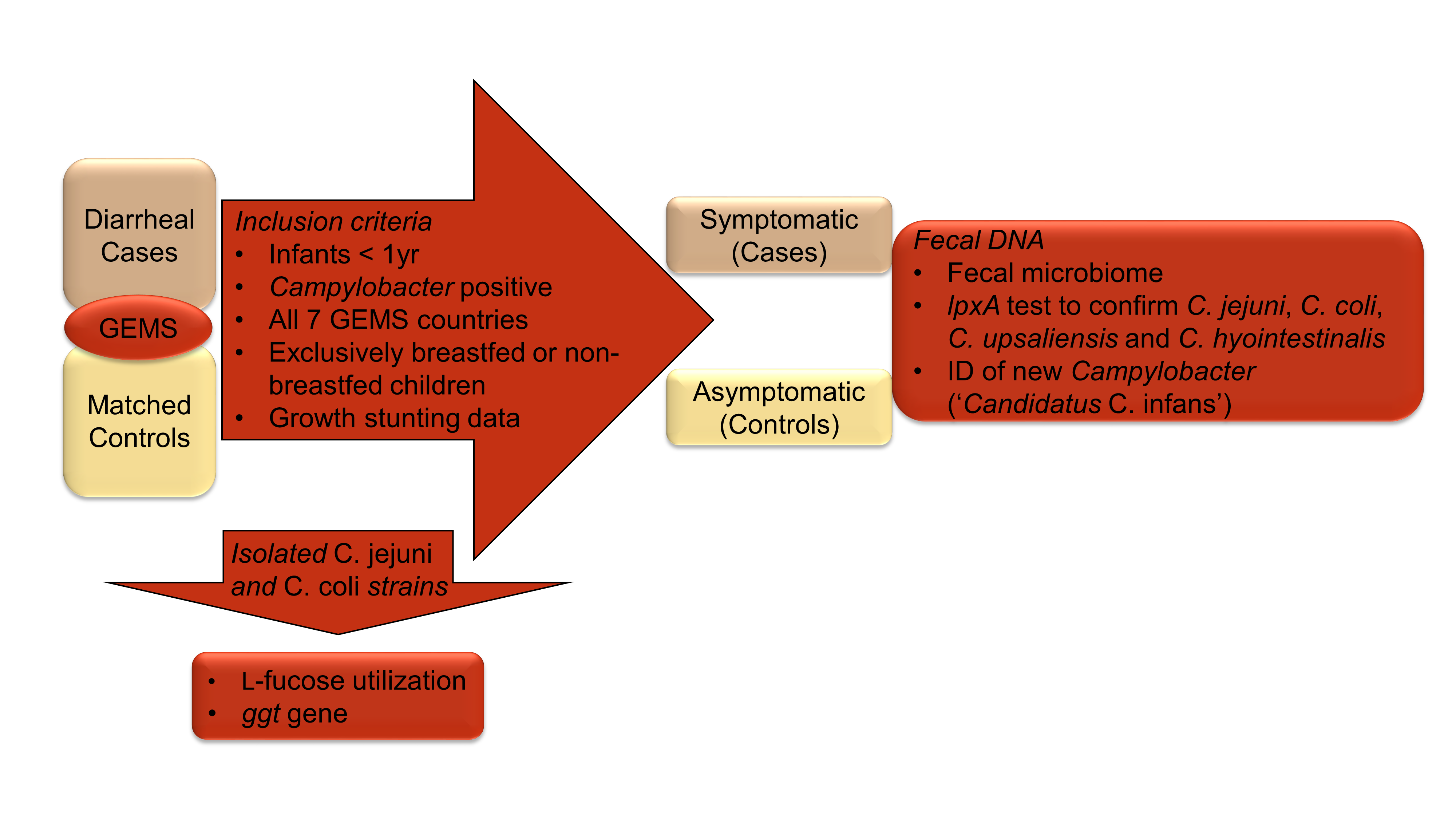

Supplement: FIG S1 [file mSphere.00735-19-sf001.tif]

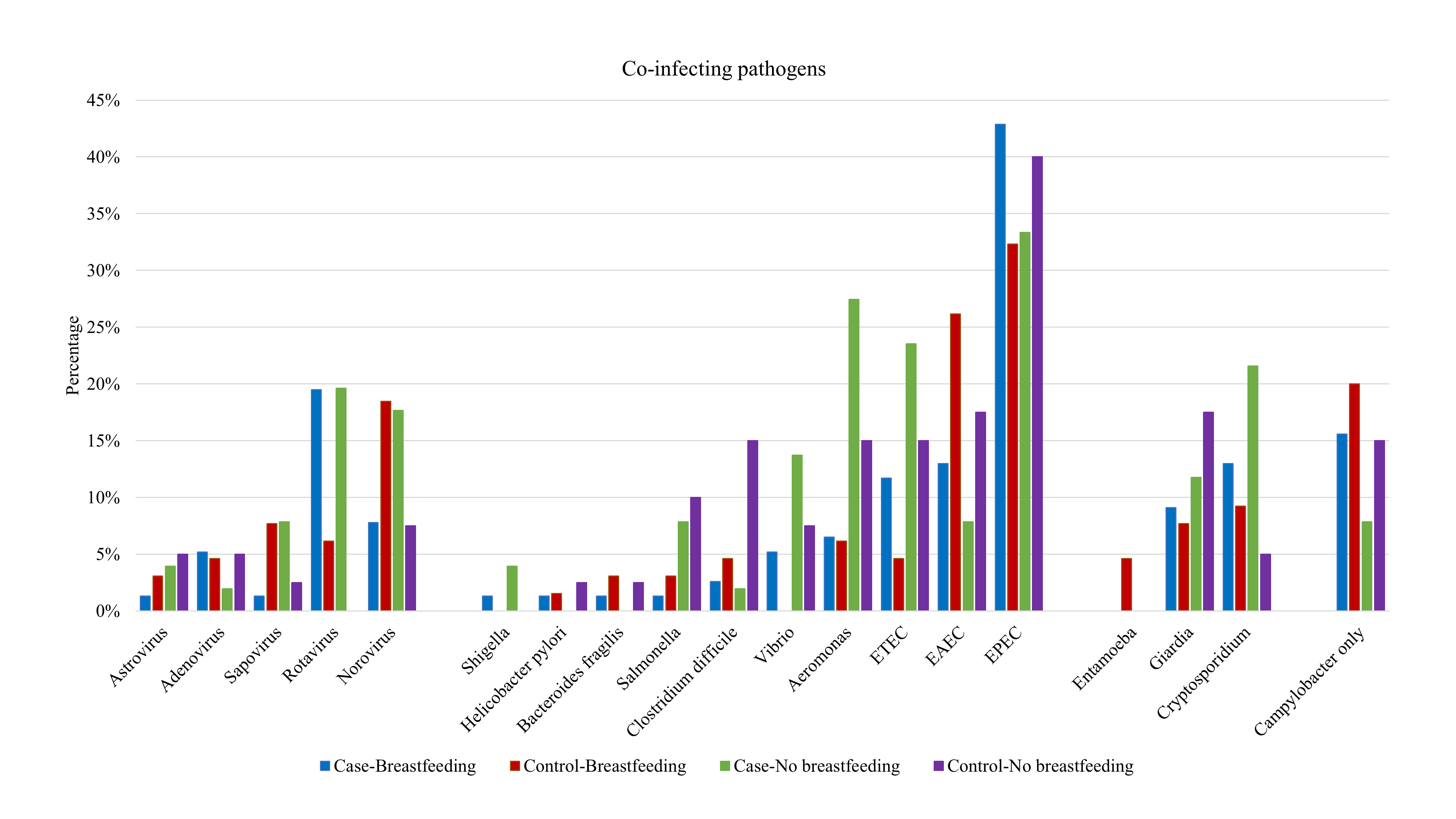

Supplement: FIG S2 [file mSphere.00735-19-sf002.tif]

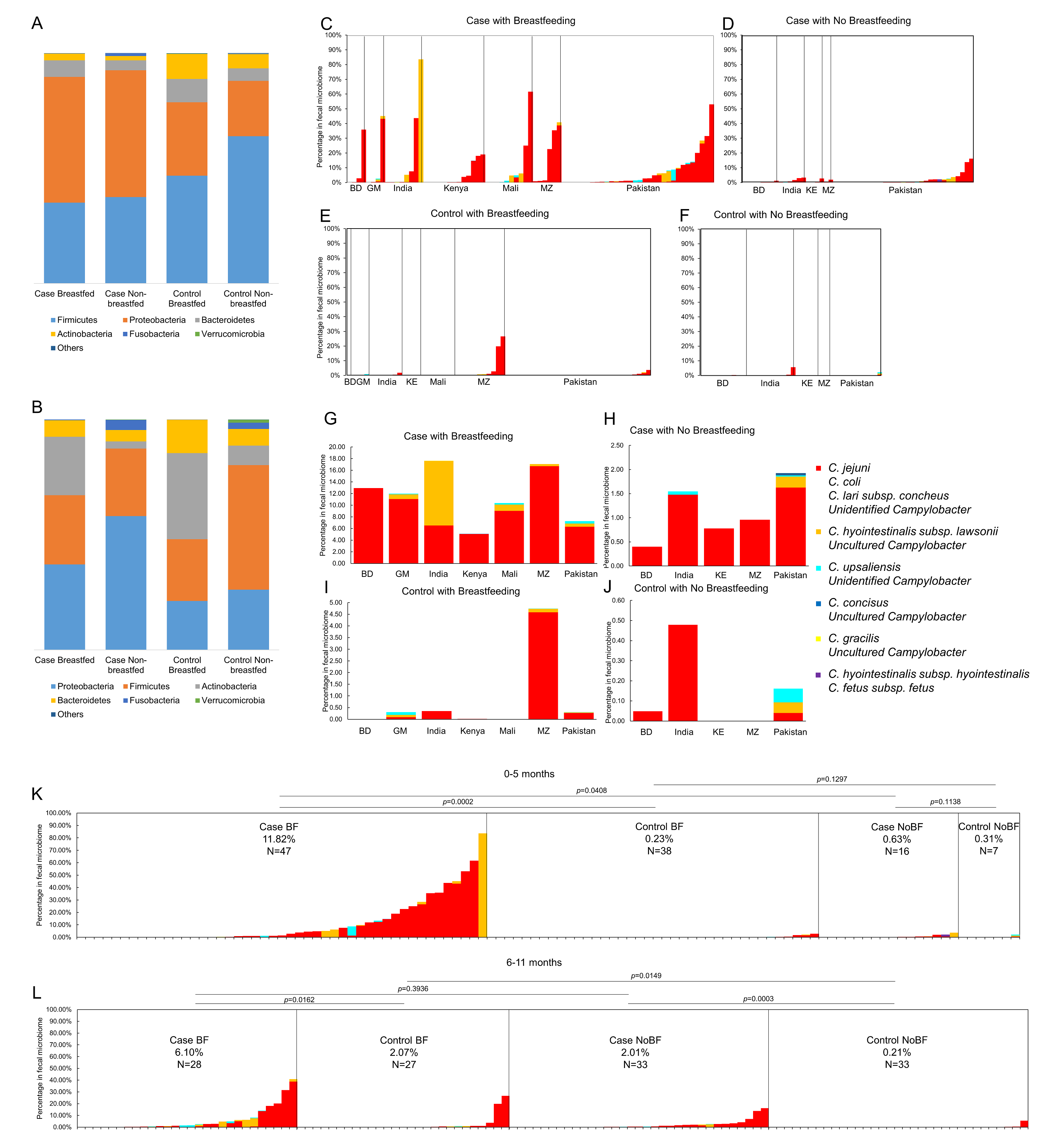

Supplement: FIG S3 [file mSphere.00735-19-sf003.tif]

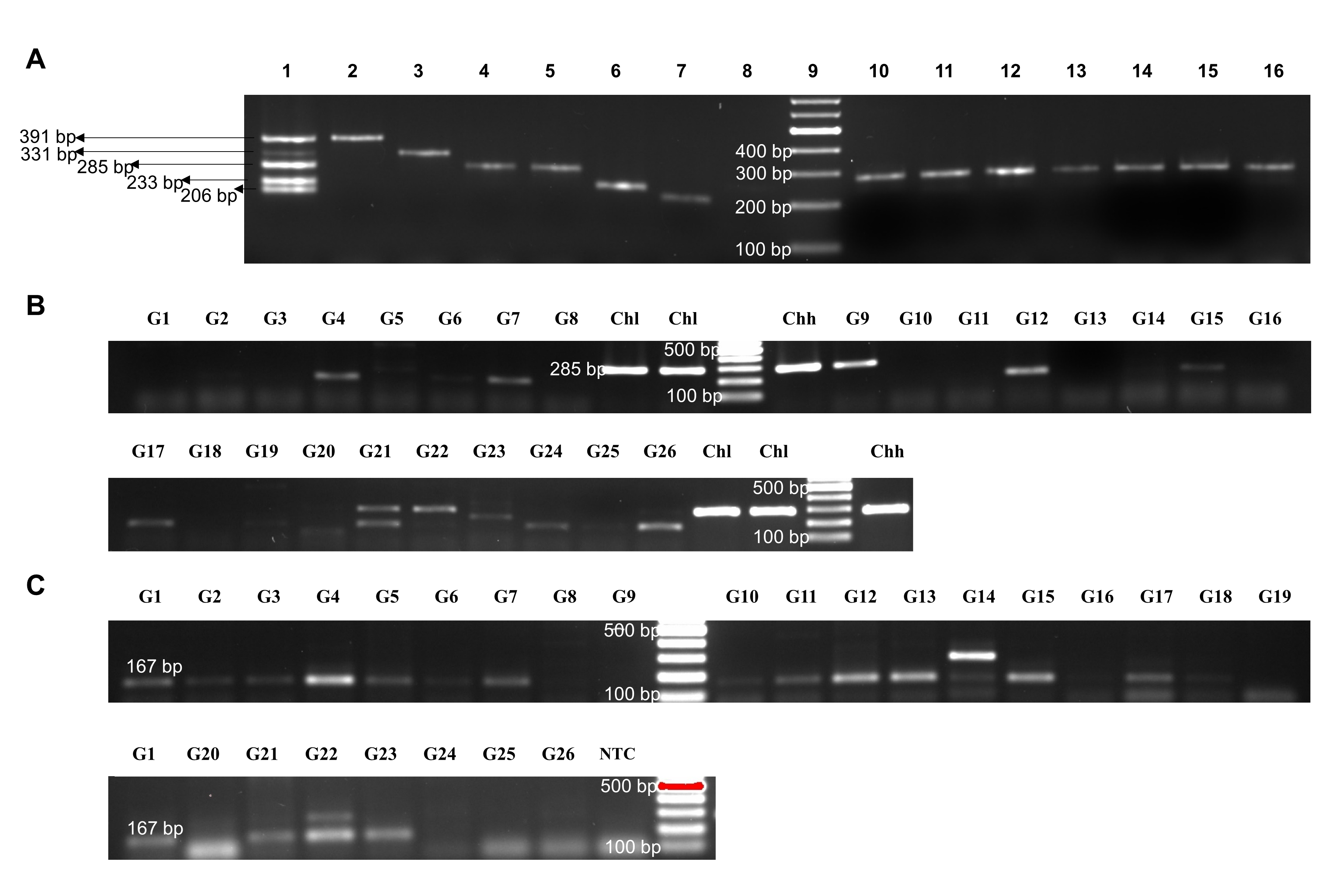

Supplement: FIG S4 [file mSphere.00735-19-sf004.tif]

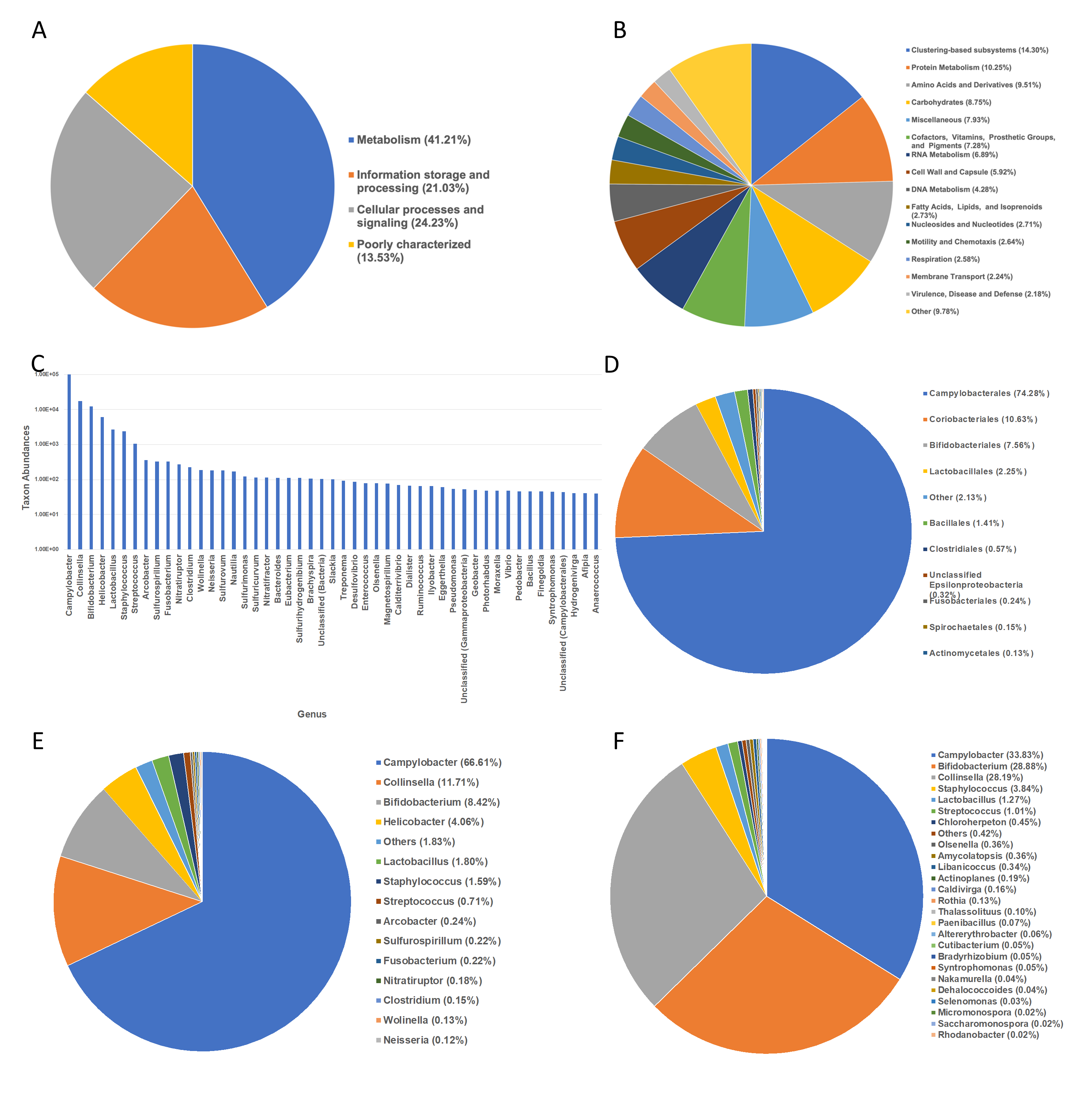

Supplement: FIG S5 [file mSphere.00735-19-sf005.tif]
