# Supplementary material for: Campylobacter Abundance in Breastfed Infants and Identification of a New Species in the Global Enterics Multicenter Study
Source: mSphere. 2020 Jan 15;5(1):e00735-19. doi: 10.1128/mSphere.00735-19 (PMC6968651; doi:10.1128/mSphere.00735-19)
Supplement: TABLE S1 [file mSphere.00735-19-st001.docx]

**Table S1**. Characteristics of study subjects with symptomatic or asymptomatic *Campylobacter* infections.

| Site | Total  (n=233) | Case  (n=128) | Control  (n=105) | BF  (n=142) | No BF  (n=91) | MSD  (n=111) | LSD  (n=17) | 0-5 month  (n=110) |  | 6-11 month  (n=123) |  |  |
| --- | --- | --- | --- | --- | --- | --- | --- | --- | --- | --- | --- | --- |
| Bangladesh | 21 | 12 | 9 | 5 | 16 | 12 | 0 | 8 |  | 13 |  |  |
| India | 34 | 14 | 20 | 15 | 19 | 12 | 2 | 16 |  | 18 |  |  |
| Kenya | 23 | 17 | 6 | 17 | 6 | 17 | 0 | 14 |  | 9 |  |  |
| Mali | 17 | 10 | 7 | 17 | 0 | 10 | 0 | 12 |  | 5 |  |  |
| Mozambique | 21 | 8 | 13 | 17 | 4 | 6 | 2 | 6 |  | 15 |  |  |
| Pakistan | 109 | 63 | 46 | 63 | 46 | 51 | 12 | 51 |  | 58 |  |  |
| The Gambia | 8 | 4 | 4 | 8 | 0 | 3 | 1 | 3 |  | 5 |  |  |

Note: Case: children with diarrhea; Control: matched children with no diarrhea; BF: breastfeeding; MSD: moderate to severe diarrhea; LSD: less severe diarrhea.
