# Supplementary material for: Campylobacter Abundance in Breastfed Infants and Identification of a New Species in the Global Enterics Multicenter Study
Source: mSphere. 2020 Jan 15;5(1):e00735-19. doi: 10.1128/mSphere.00735-19 (PMC6968651; doi:10.1128/mSphere.00735-19)
Supplement: TABLE S2 [file mSphere.00735-19-st002.docx]

**Table S2**. Significantly different genera between cases and controls*

| **Breastfeeding** | Case (%) | Control (%) | *p*-values (corrected) |
| --- | --- | --- | --- |
| *Collinsella* | 0.56 | 3.19 | 1.05×10^-5^ |
| *Bifidobacterium* | 1.46 | 3.78 | 3.90×10^-5^ |
| *Coriobacteriaceae* uncultured species | 0.64 | 3.49 | 8.53×10^-5^ |
| *Bacteroides* | 0.47 | 4.81 | 0.0016 |
| *Enterococcus* | 0.60 | 2.96 | 0.0099 |
| *Dorea* | 0.03 | 0.29 | 0.0117 |
| *Ruminococcus gnavus* group | 0.32 | 0.60 | 0.0126 |
| *Intestinibacter* | 0.01 | 0.15 | 0.0192 |
| *Actinomyces* | 0.01 | 0.10 | 0.0221 |
| *Faecalibacterium* | 0.40 | 0.54 | 0.0221 |
| *Megasphaera* | 0.32 | 3.17 | 0.0307 |
| *Blautia* | 0.14 | 1.00 | 0.0436 |
| **No-breastfeeding** | Case (%) | Control (%) | *p*-values (corrected) |
| *Escherichia-Shigella* | 48.14 | 17.05 | 0.0003 |
| *Blautia* | 0.37 | 4.47 | 0.0003 |
| *Bifidobacterium* | 0.29 | 0.81 | 0.0006 |
| *Lachnospiraceae NC2004 group* | 0.04 | 0.29 | 0.0012 |
| *Erysipelatoclostridium* | 0.14 | 0.57 | 0.0012 |
| *Lachnoclostridium* | 0.13 | 0.83 | 0.0015 |
| *Dorea* | 0.25 | 0.77 | 0.0015 |
| *Intestinibacter* | 0.02 | 0.28 | 0.0015 |
| *Eggerthella* | 0.00 | 0.19 | 0.0015 |
| *Ruminococcus gnavus group* | 0.89 | 3.45 | 0.0017 |
| *Anaerostipes* | 0.10 | 1.94 | 0.0025 |
| *Butyricicoccus* | 0.05 | 0.15 | 0.0035 |
| *Fusicatenibacter* | 0.01 | 0.18 | 0.0054 |
| *Eubacterium hallii group* | 0.01 | 0.52 | 0.0061 |
| *Lachnospiraceae* NK4A136 *group* | 0.00 | 0.50 | 0.0097 |
| *Klebsiella* | 1.17 | 2.29 | 0.0102 |
| *Ruminiclostridium* 5 | 0.31 | 0.96 | 0.0118 |
| *Clostridium sensu stricto* 1 | 0.14 | 1.28 | 0.0148 |
| *Lachnospiraceae* uncultured *species* | 0.01 | 0.15 | 0.0158 |
| *Coprococcus* 1 | 0.01 | 0.08 | 0.0175 |
| *Coprococcus* 3 | 0.03 | 0.14 | 0.0203 |
| *Prevotella* 9 | 1.10 | 2.59 | 0.0227 |
| *Lactococcus* | 0.09 | 0.58 | 0.0238 |
| *Ruminococcaceae* UCG-002 | 0.00 | 0.15 | 0.0310 |
| *Faecalibacterium* | 0.77 | 2.41 | 0.0445 |

**Campylobacter* not included. Grey shading indicates which group is increased.
