# Supplementary material for: Campylobacter Abundance in Breastfed Infants and Identification of a New Species in the Global Enterics Multicenter Study
Source: mSphere. 2020 Jan 15;5(1):e00735-19. doi: 10.1128/mSphere.00735-19 (PMC6968651; doi:10.1128/mSphere.00735-19)
Supplement: TABLE S3 [file mSphere.00735-19-st003.docx]

Table S3. Correlations between *Campylobacter* and gut microbes.

|  | Campy | ***Dorea*** | ***Blautia*** | **Kleb** | **Erysip** | Enterob | ***Faecal*** | *Enteroc* | *Collin* | ***Rumino*** | **Lacto** | **Anaero** |
| --- | --- | --- | --- | --- | --- | --- | --- | --- | --- | --- | --- | --- |
| ***Dorea*** | -0.28 |  |  |  |  |  |  |  |  |  |  |  |
| ***Blautia*** | -0.27 | 0.64 |  |  |  |  |  |  |  |  |  |  |
| **Kleb** | -0.24 |  |  |  |  |  |  |  |  |  |  |  |
| **Erysip** | -0.23 | 0.11 | 0.30 | 0.21 |  |  |  |  |  |  |  |  |
| Enterob | -0.22 |  |  | 0.76 | 0.20 |  |  |  |  |  |  |  |
| ***Faecal*** | -0.21 | 0.50 | 0.53 |  | 0.13 |  |  |  |  |  |  |  |
| *Enteroc* | -0.21 |  |  | 0.14 | 0.28 | 0.18 |  |  |  |  |  |  |
| *Collin* | -0.19 | 0.38 | 0.41 | -0.16 |  | -0.15 | 0.37 |  |  |  |  |  |
| ***Ruminoc*** | -0.19 |  | 0.16 | 0.22 | 0.53 | 0.24 | 0.14 | 0.28 |  |  |  |  |
| **Lacto** | -0.19 |  |  | 0.39 | 0.21 | 0.42 |  |  |  | 0.25 |  |  |
| **Anaero** | -0.18 | 0.40 | 0.40 |  | 0.12 |  | 0.36 |  | 0.19 |  |  |  |

Note: Pseudo *p*-values ranged from 0.00-0.03 for all shown so only significant coefficients of correlation are shown. Names in **bold** are decreased in non-breastfed children with diarrhea compared to control, Names in *italics* are decreased in breastfed children with diarrhea compared to control. Campy: *Campylobacter*, Kleb: *Klebsiella*, Erysip: *Erysipelatoclostridium*; Enterob: *Enterobacter*; Faecal: *Faecalibacterium*; Enteroc: *Enterococcus*; Collin: *Collinsella*; Rumino: *Ruminococcus* *gnavus* group; Lacto: *Lactococcus*; Anaero: *Anaerostipes*
