# Supplementary material for: Campylobacter Abundance in Breastfed Infants and Identification of a New Species in the Global Enterics Multicenter Study
Source: mSphere. 2020 Jan 15;5(1):e00735-19. doi: 10.1128/mSphere.00735-19 (PMC6968651; doi:10.1128/mSphere.00735-19)
Supplement: TABLE S4 [file mSphere.00735-19-st004.docx]

**Table S4**. Coinfection of *Campylobacter* species in study subjects detected by 16S rRNA sequencing and confirmed by *lpxA* multiplex PCR.

| *Campylobacter* infections | All | Case +BF | Case -BF | Control +BF | Control -BF |
| --- | --- | --- | --- | --- | --- |
| *C. jejuni* | 74 | 35 | 23 | 13 | 3 |
| *C. coli* | 8 | 1 | 2 | 3 | 2 |
| ‘*Candidatus* C. infans’ | 8 | 3 | 2 | 3 | 0 |
| *C. upsaliensis* | 6 | 1 | 2 | 1 | 2 |
| *C. jejuni + C. coli* | 12 | 3 | 2 | 6 | 1 |
| *C. jejuni +* ‘*Candidatus* C. infans’ | 7 | 5 | 1 | 1 | 0 |
| *C. jejuni + C. upsaliensis* | 5 | 5 | 0 | 0 | 0 |
| *C.jejuni + C.hyointestinalis* subsp. *hyointestinalis* | 1 | 0 | 1 | 0 | 0 |
| ‘*Candidatus* C. infans’*+ C. upsaliensis* | 2 | 0 | 1 | 1 | 0 |
| *C. jejuni + C. coli +* ‘*Candidatus* C. infans’ | 3 | 3 | 0 | 0 | 0 |
| *C. jejuni + C. coli + C. upsaliensis* | 1 | 0 | 0 | 1 | 0 |
| *C. jejuni +* ‘*Candidatus* C. infans’*+ C. upsaliensis* | 4 | 3 | 0 | 0 | 1 |
| Total | 131 | 59 | 34 | 29 | 9 |

BF: exclusive breastfeeding
